# Mollugin reacts with phenol thiol but not produce modification on cysteine discovered by a phenol thiol probe

**DOI:** 10.3389/fcell.2025.1629762

**Published:** 2025-08-20

**Authors:** Tao Liu, Ruibing Qi, Wanshi Feng, Zhuohao Li, Zerun Zhong, Chuan Bai

**Affiliations:** ^1^ Department of Pathogen Biology and Biosecurity, Zhongshan School of Medicine, Sun Yat-sen University, Guangzhou, China; ^2^ Institute of Human Virology, Guangzhou, China; ^3^ Faculty of Forensic Medicine, Guangdong Province Translational Forensic Medicine Engineering Technology Research Center, Guangzhou, China

**Keywords:** nucleophilic chemical probe, Mollugin, cysteine, natural products, covalent modification

## Abstract

Electrophilic compounds from natural products (NPs) and metabolites can covalently modify the cysteines of target proteins to induce biological activities. To facilitate the discovery of novel NPs and metabolites, chemical probes with various thiol groups—mimicking the reactivity of cysteine—have been developed. These probes are designed to react with electrophilic groups of NPs and metabolites in an electrophilic addition mechanism, with the resulting adducts having molecular masses which equal to the sum of the probe and the target compound. This principle has been fundamental to analyzing mass spectrometry (MS) data and calculating the exact molecular weights of the target compound. In this study, we report a phenol thiol probe initially designed to mimic cysteine reacts with Mollugin and other structurally related NPs in an electrophilic free radical addition mechanism, and thus leads to the incorporation of not only the thiol probe but also a hydroxyl group in the adducts. Our results demonstrate that the phenol thiol group of the probe cannot always represent the thiol in cysteine to discover novel NPs or metabolites that can covalently modify cysteines.

## 1 Introduction

**FIGURE 1:**
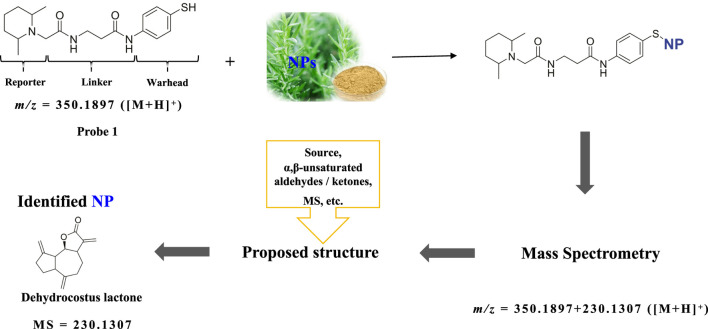
Working mechanism of **Probe 1** and rationale of the deduction of the molecular weight of the NP. **Probe 1** reacts with the electrophilic NP in the NP extract. The accurate molecular mass of the product can be detected from high resolution MS1, from which the molecular weight and molecular formula can be calculated. Dehydrocostus lactone is employed as an example.

Natural product (NP) or metabolite compounds containing electrophilic groups can covalently modify the cysteines of target proteins to induce biological activities (Berdan et al., 2019; Gehrtz and London, 2021; Yang et al., 2012; Hoch et al., 2020; Qin et al., 2020; Byun et al., 2023). Given the scarcity and high chemical diversity of such compounds, a variety of chemical probes have been developed to facilitate the discovery and identification of novel electrophilic NPs or metabolites from extracts of cells or body fluids (Hughes, 2021; Wesche et al., 2017; Reimer and Hughes, 2017; Castro-Falcón et al., 2016; Jiang et al., 2020; Weerapana et al., 2010). The key part of these probes is the “war head”, which is designed to react with the electrophilic groups of the target compounds. We previously reported a nucleophilic chemical probe (**Probe 1**) with a phenol thiol group as the “war head” for the discovery of novel electrophilic NPs as cysteine modulators (Figure 1) (Gao et al., 2022). This chemical probe can afford accurate mass information of NPs, by assuming that the phenol thiol group is added to the NP and the molecular weight of the product is the sum of the molecular weights of the probe and NP. This mechanism also has been successfully applied to other chemical probes that employ alky thiol or cysteine as the “war head”.

We applied **Probe 1** to discover new electrophilic NPs from various source of NPs such as the fruit stalk of *Pedicellus melo*, the fruit body of *Ganoderma lucidum*, gamboge (resin from *Garcinia hanburyi*), the flower of *Chrysanthemum morifolium Ramat*, cocklebur, and the leaf of Ginkgo *biloba*. We identified some electrophilic NPs that have been reported such as cucurbitacin B, gambogic acid A and xanthatin. We also successfully discovered a novel NP **BG-1**,then clarified its structure and identified potential target proteins (Gao et al., 2022). These successful results showed the efficacy of the assumption on the calculation of target NP’s accurate molecular weight. However, in our recent effort to identify electrophilic NPs from *Rubia cordifolia* L. (madder), we found a new type of addition reaction of **Probe 1** with some NPs that did not follow the assumption above. More importantly, we discovered that **Probe 1** did not always capture the NPs that can react with cysteine. These “off-target” effects showed that it is necessary to carefully verify the accuracy of “war heads” of our and other chemical probes.

## 2 Materials and methods

### 2.1 Materials and reagents

We used commercially available reagents and solvents without further purification. Preparative thin-layer chromatography (TLC) silica gel plates (HSGF254) were purchased from Yantai Jiangyou Silica Gel Development Co., Ltd., with a coating thickness of 0.4–0.5 mm and a pH range of 6.2–6.8. TLC visualization was performed under UV light (254 nm or 365 nm) or by phosphomolybdic acid staining. Flash chromatography was carried out using 300–400 mesh silica gel. The following reagents were used: ethanol, ascorbic acid, methanol (HPLC grade), dimethyl sulfoxide (DMSO), dichloromethane (DCM), 3,5-dimethylphenol thiol (DPT), tetrahydrofuran (THF), N,N-dimethylformamide (DMF), tert-butyl hydroperoxide (TBHP), 2,2,6,6-tetramethylpiperidin-1-oxyl (TEMPO), Glabridin, and various phenol thiol and small thiol molecules for chemical synthesis. All reagents were purchased from commercial suppliers including Shanghai Macklin Biochemical Co., Ltd., Anhui Zesheng Technology Co., Ltd., Shanghai Titan Scientific Co., Ltd., and Shanghai TCI Development Co., Ltd., and used as received. *Rubia cordifolia* L. (Rubiaceae) was purchased from Anguo Medicine Source Trading Co., Ltd. (MoYuan Medicinal Materials) and authenticated by Dr. Peng Guangtian of the Department of Medicinal Botany, College of Chinese Materia Medica, Guangzhou University of Chinese Medicine. The *R. cordifolia* extract was prepared in-house. Mollugin was obtained from Baoji Herbest Bio-Tech Co., Ltd. (batch number HR4014W5, purity ≥98%). HPLC analysis by Herbest Bio-Tech confirmed a retention time (RT = 7.44 min) consistent with the reference standard and a purity of 99.6%, meeting enterprise specifications.

### 2.2 The general methods of the reaction of probe 1 with NPs or natural extract

The NP (natural extract) (10 mg), ascorbic acid (2 mg) and Probe 1 (3 mg) was dissolved in certain solvent (2 mL, methanol, DCM or DMF depending on the solubility of NP or natural extract) in a flask. The reaction was monitored with TLC. After TLC showed that the NP spot disappeared (or after reacting with the natural extract for 24 h), the reaction solvent was removed with vacuum and the residue was stored in −40°C as analysis samples. Right before the analysis by liquid chromatograph mass spectrometer (LC-MS^n^), the sample was dissolved with methanol to afford a 20 μg/mL (20 ppm) solution in the total weight of NP, **probe 1** and ascorbic acid.

### 2.3 The general methods of chromatography and mass spectrometry

LC-MS/MS analysis was performed with a Q Exactive mass spectrometer (Thermofisher Scientific, United States), equipped with electrospray sources and Dionex UltiMate 3000 UHPLC (Thermofisher Scientific, United States). Aqueous mobile phase (A) was 0.1% formic acid in water and organic mobile phase (B) was acetonitrile. Samples (2 µL) were analyzed with a Hypersil GOLD-C18 HPLC column (2.1 × 100 mm, 1.9 µm) (Thermofisher Scientific, United States) with mobile phase in flow rate of 0.3 mL/min. The details of the HPLC gradient and the settings of mass spectrometry were described in support information.

### 2.4 Preparation method of *Rubia cordifolia* L. Extract

*Rubia cordifolia* L. raw material (500 g) was macerated with 1 L of 95% (v/v) ethanol at room temperature for 72 h. The resulting ethanol extract was concentrated to a semi-solid state using a rotary evaporator, and residual ethanol was completely removed by vacuum drying. The obtained extract was aliquoted into airtight amber vials and stored at −40°C until use.

### 2.5 Isolation and purification of compound 5 from extract of *Rubia cordifolia* L

Ethanol extract of *R. cordifolia* L. (500 mg) was dispensed in 60% methanol water solution (7 mL) and the solution was loaded to a C-18 column (40 g) and purified with gradient (12 mL/min) described in Supplementary Table S1. The eluted fractions were tested with high resolution mass spectrometry to identify the fractions containing the targeted molecular weight (*m/z* = 285.1117 [M + H]^+^). These fractions were collected and the solvent was removed to afford light brown solid. The obtained solid further purified with silica gel column to afford pure compound **5** (3.2 mg).

### 2.6 Method for reacting Mollugin or Glabridin with various phenol thiols or thiols

Mollugin or Glabridin (1 equivalent) was dissolved in 3–5 mL of methanol/dichloromethane/tetrahydrofuran/DMF together with phenol thiol or thiol compound (1.2 equivalent), and the mixture was stirred at room temperature. The reaction progress was monitored by TLC. Upon completion of the reaction, the solvent was removed by rotary evaporation under reduced pressure, and the residue was purified by silica gel column chromatography. The purified product was structurally confirmed by LC-MS, NMR, or MS.

## 3 Results

### 3.1 Probe 1 reacts with Mollugin through an unexpected mechanism

**FIGURE 2:**
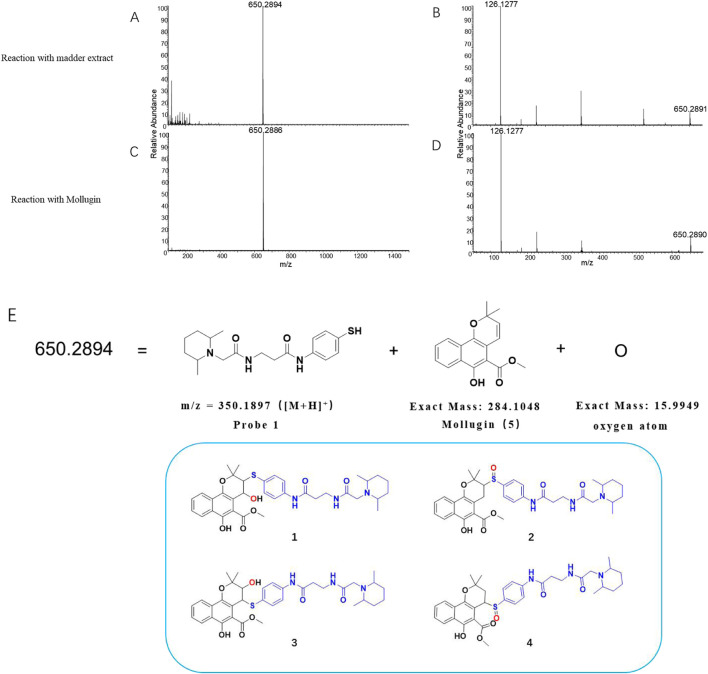
Identification of the effective signal at *m/z* = 650.2894. **(A)** Madder extract reacted with **Probe 1**, showing the parent ion with *m/z* = 650.2894 in MS^1^. **(B)** Madder extract reacted with **Probe 1**, showing the parent ion with *m/z* = 650.2894 in MS^2^. **(C)** Mollugin standard reacted with **Probe 1**, showing the parent ion with *m/z* = 650.2886 in MS^1^. **(D)** Mollugin standard reacted with Probe 1, showing the parent ion with *m/z* = 650.2886 in MS^2^. **(E)** Possible structures of the compounds containing **Probe 1** (blue), Mollugin (black), and an oxygen atom (red), corresponding to the parent ion with *m/z* = 650.2894.

**SCHEME 1:**
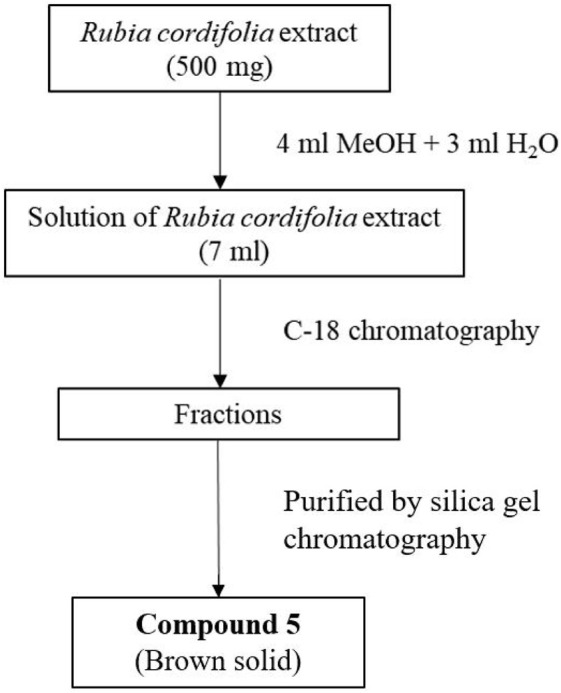
Isolation and purification process of compound **5** from *Rubia cordifolia* L.

In this study, we utilized **Probe 1** to discover cysteine-targeted NPs from *R. cordifolia* L. (madder), a traditional medicinal plant widely used in Asia (Wen et al., 2022). The reaction was monitored by LC-MS as previously described (Supplementary Tables S1, S2). A qualified adduct signal with *m/z* = 650.2894 was detected (Figures 2A,B) and we deduce the expected exact mass of the corresponding NP with the established principle. However, this mass did not match with any known NPs previously reported from *R. cordifolia* L. Intriguingly, the observed mass could be rationalized if an additional oxygen atom was incorporated into the product formed from the reaction of **Probe 1** with Mollugin, a well-documented bioactive compound from *R. cordifolia* L. (Wen et al., 2022; Zhu et al., 2013). To validate this hypothesis, we reacted a Mollugin standard with **Probe 1** and observed an identical LC-MS signal to that obtained from the *R. cordifolia* L. extract (Figures 2A–D). Subsequent isolation of the NP (compound **5**) from the extract of *R. cordifolia* L. was performed (Scheme 1), and it was confirmed as Mollugin via MS^n^ and NMR (Nuclear Magnetic Resonance) (Supplementary Figures S1–S6). We also confirmed the structure of the adduct of Mollugin and **Probe-1** with MS^2^ (Supplementary Figure S7). We proposed potential structures for the reaction product as compounds **1–4** (Figure 2E). These results demonstrated that the phenol thiol group of **Probe 1** reacts with Mollugin through a mechanism distinct from the previously established one.

### 3.2 Elucidation of the structure of the reaction product and that Mollugin does not react with cysteine

**FIGURE 3:**
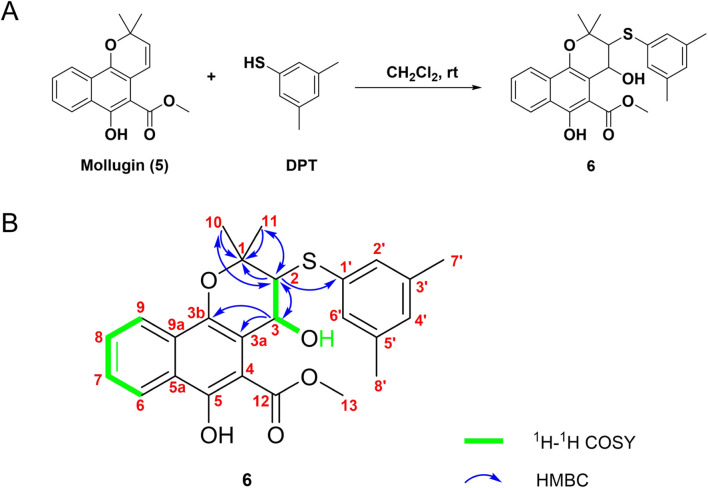
Confirmation of the structure of compound **6**. **(A)** Mollugin reacts with DPT. **(B)** Structure of compound **6** and correlation of with ^1^H and ^13^C NMR data.

**FIGURE 4:**
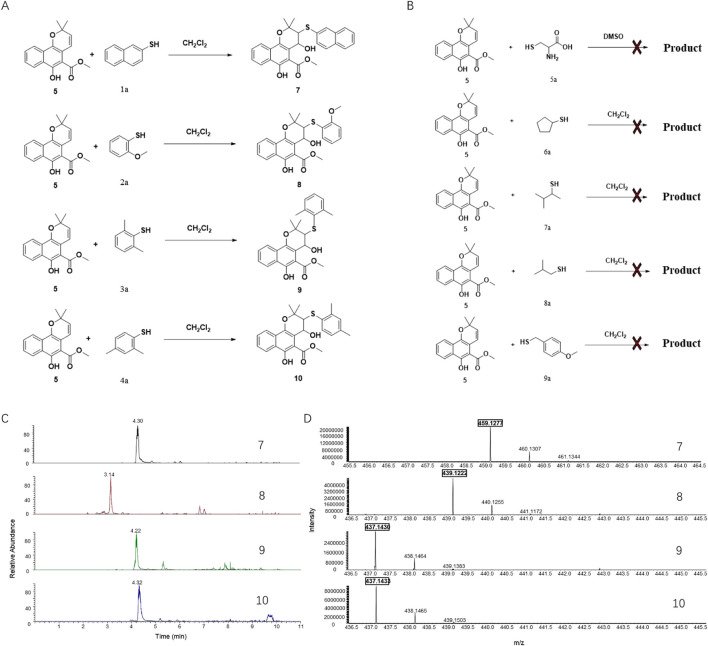
Mollugin does not react with various alkyl thiols but reacts with various phenol thiols, and the products demonstrate the addition of a hydroxyl group. **(A)** Mollugin can react with various phenol thiols. **(B)** Mollugin does not react with various alkyl thiols. **(C)** Chromatograms of the products (compounds **7–10**) derived from the reactions of Mollugin with phenol thiols. **(D)** MS spectra of the products (compounds **7–10**) derived from the reactions of Mollugin with phenol thiols.

Subsequently, Mollugin was reacted with 3,5-dimethyl phenol thiol (DPT), as a surrogate for Probe 1, and the structure of the product was elucidated (Figure 3). Comprehensive analytical characterization of the product was conducted (Supplementary Figures S8–S15). These data enabled detailed assignment of the proton (H) and carbon (C) signals for compound 6, which were systematically compared with those of Mollugin (Table 1). Analysis of the H-H and C-H correlation spectra from 2D NMR revealed the addition of a phenol thiol group to the C3 carbon of the alkene moiety in compound **6** (Figure 3B). In addition to DPT, other phenol thiols reacted with Mollugin under mild conditions (25°C, catalyst-free), yielding the corresponding products (Figure 4A). Notably, Mollugin exhibited no reactivity toward alkyl thiols, including cysteine, under the same conditions (Figure 4B). The formation of compounds **7–10** was confirmed by LC-MS, with observed molecular weights consistent with the expected structures (Figures 4C,D). This distinct selectivity toward phenol thiols demonstrated that Mollugin could not modulate cysteine as initially expected, highlighting its unique reactivity profile.

**TABLE 1:** H and C attribution and comparison between compounds **5** and **6**.

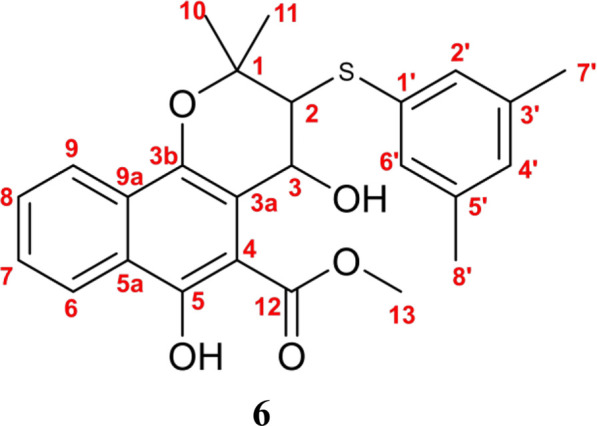			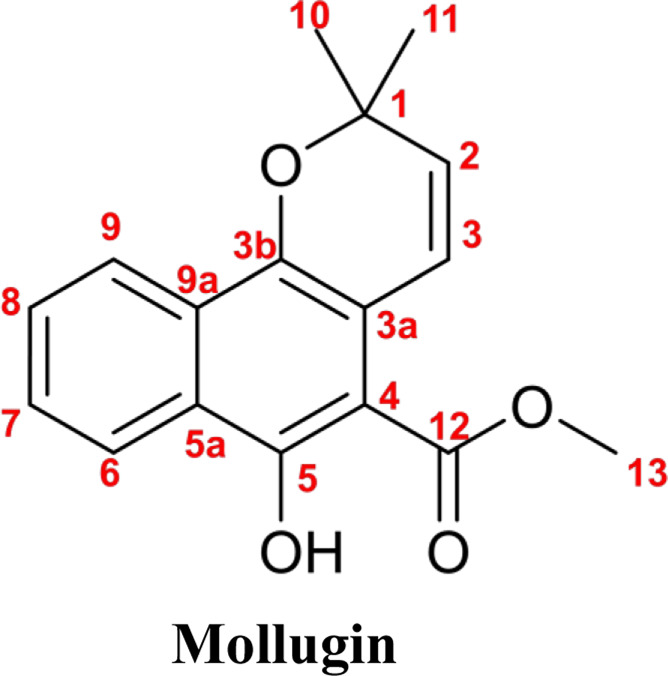	
No.	Compound 6		Mollugin	
	δC	δH(J/Hz)	δC	δH(J/Hz)
1	79.00	No	75.41	no
2	61.02	3.50 (d, J = 8.5 Hz, 1 H),	130.36	5.80 (d, J = 10.0 Hz, 1 H)
3	68.27	5.26 (d, J = 8.4 Hz, 1 H)	121.57	6.87 (d, J = 10.0 Hz, 1H)
3a	112.92	No	112.87	No
3b	141.27	No	141.21	No
4	104.62	No	106.20	No
5	155.14	No	151.84	No
5a	125.62	No	125.14	No
6	123.84	8.46 – 8.33 (d, 1 H)	123.84	8.21 (d, J = 8.4 Hz, 1 H)
7	126.89	7.55 - 7.61 (m, 1 H)	127.01	7.56 (t, J = 7.6 Hz, 1 H)
8	129.04	7.68 – 7.61 (m, 1 H)	129.42	7.64 (t, J = 7.5 Hz, 1 H)
9	122.34	8.25 – 8.14 (d, 1 H)	121.96	8.06 (d, J = 8.3 Hz, 1 H)
9a	128.66	No	127.83	No
10	27.91	1.65 (s, 3 H)	27.05	1.42(s,6 H)
11	20.72	1.53 (s, 3 H)		
12	171.58	No	170.58	No
13	52.59	4.04 (s, 3 H)	53.06	3.98(s,3 H)
1'	135.31	No		
2'	128.90	7.18 (s, 2 H)		
6'				
3'	138.82	No		
5'		No		
4'	129.25	6.89 (s, 1 H)		
7'	21.19	2.30 (s, 6 H)		
8'				
-OH(C3)		3.31 (s, 1 H)		
-OH(C5)		11.19 (s, 1 H)		11.21 (s, 1 H)

### 3.3 The proposed radical reaction mechanism and its validation

**SCHEME 2:**
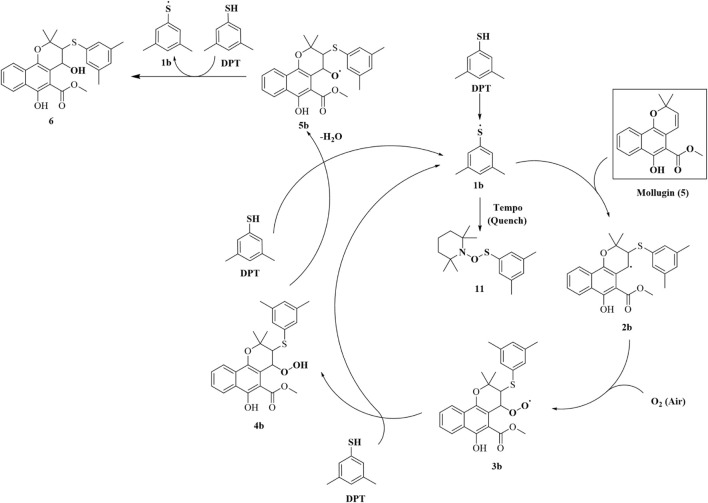
Proposed free radical mechanism.

**FIGURE 5:**
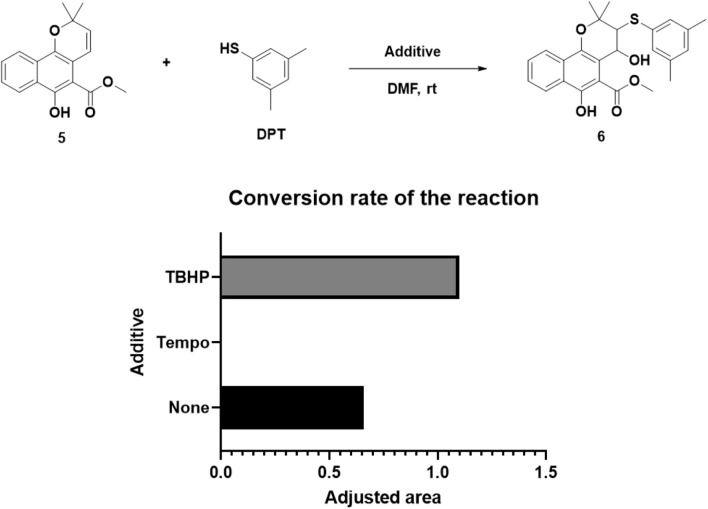
The influence of various additives on the reaction conversion rate was quantified by analyzing the chromatographic peak area of the target product. The adjusted area is defined as the peak area ratio of the reaction product to the internal standard.

**SCHEME 3:**
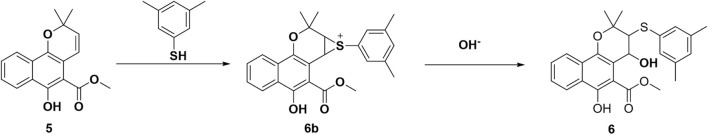
Proposed sulfonium-based mechanism.

**FIGURE 6:**
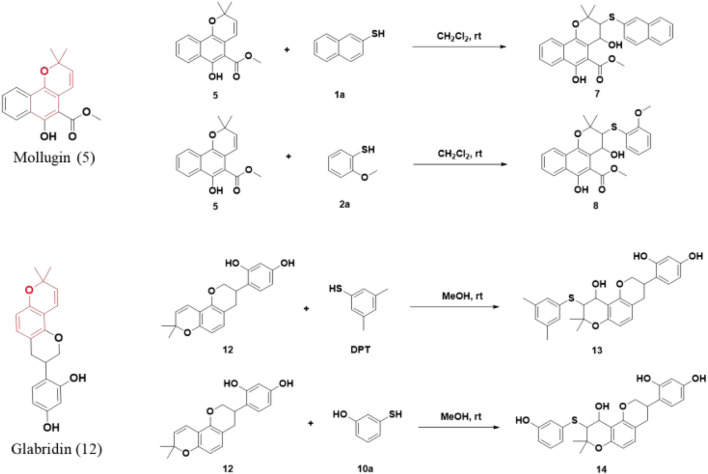
Chemical formulas for the reactions of Mollugin or Glabridin with various phenol thiols.

The addition of both a phenol thiol group and a hydroxyl group to the alkene bond of Mollugin is not a common electrophilic reaction in which typically only the nucleophilic molecule is added to the electrophilic NP without concomitant hydroxylation. This observation led us to propose a free radical addition mechanism, which has been reported to result in hydroxyl group addition (Scheme 2) (Zhou et al., 2015; Choudhuri et al., 2018; Wang et al., 2016). First, the clear selectivity toward phenol thiols over alky thiols aligns with the known propensity of phenol thiols to generate radicals under mild conditions, even without of strong radical inducers, a reactivity not typically observed with alkyl thiols. Second, free radical scavenger TEMPO and initiator TBHP can effectively abolish or facilitate this reaction (Figure 5; Supplementary Figures S16, 17) and the adduct of TEMPO and DPT was also isolated (Supplementary Figures S19–S21). Third, we ruled out a sulfonium-based mechanism (Taniguchi, 2006), in which the phenol thiol would form a sulfonium intermediate with the alkene bond of Mollugin, followed by nucleophilic attack by a hydroxyl group to yield the final product (Scheme 3). The other nucleophilic bromide anion did not afford any expected addition product, showing that the sulfonium is unlikely to be involved in this reaction (Supplementary Figure S18). Finally, we demonstrated that Glabridin, a NP containing the same reactive moiety as Mollugin, undergoes a similar reaction with phenol thiol (Figure 6). The structure of the corresponding synthetic product has been confirmed by NMR and MS (Supplementary Figures S22–S33). This observation suggests that the reaction mechanism is not unique to Mollugin but is likely applicable to a broader range of NPs with diverse structures.

## 4 Discussion

Chemical probes capable of reacting with specific functional groups have been employed as powerful tools in the discovery of novel compounds from NPs and metabolites. The reaction between the “war head” of these probes and the functional groups in target compounds is vital for obtaining the key information, such as the accurate molecular weight that can be used to deduce the molecular formula of the target compound. However, the chemical properties of the “war head” can only partially represent those of the desired molecules. In this study, we utilized a phenol thiol group as the “war head” in **Probe 1**, based on the assumption that it could mimic the reactivity of the thiol group in cysteine. Our unpublished studies of **Probe 1** showed that its phenol thiol group exhibits higher nucleophilic reactivity than the alky thiol group toward electrophilic groups such as unsaturated carbonyls. Interestingly, here we observed that the phenol thiol “war head” reacted with Mollugin, whereas cysteine was unreactive under the same conditions. This discrepancy suggests that **Probe 1** may yield false-positive results in the discovery of cysteine-targeting NPs. Furthermore, Mollugin afforded an unexpected product incorporating **Probe 1** together with a hydroxyl group. This result emphasized the necessity of carefully interpreting raw MS data to avoid erroneous conclusions regarding the molecular masses of target compounds.

Mollugin has been reported as one of the bioactive compounds in *R. cordifolia* L., exerting its effects through mechanisms related with antioxidant pathways such as the Keap1–Nrf2 axis (Liu et al., 2023). This has led to the hypothesis that Mollugin may interact with key cysteine residues in Keap1. However, our findings demonstrate that Mollugin does not react with cysteine, suggesting that its mechanism of action is unlikely to be direct modification of cysteine residues of target proteins. These results indicated that the compounds discovered by **Probe-1** may not conjugated with cysteine residuals. Instead, the high selectivity of Mollugin toward phenol thiols implies that it can be developed as a selective “war head” for targeting biologically relevant phenol thiols (Deaton et al., 2008; Hoyle et al., 2010), while avoiding the interference from alkyl thiols such as from cysteine or glutathione.

## 5 Conclusion

In conclusion, we discovered that Mollugin reacted with **Probe 1** through free radical mechanism, affording the addition product in which the hydroxyl and thiol groups of **Probe 1** were added to the alkene group of Mollugin. The chemical selectivity of Mollugin toward phenol thiols over cysteine showed that carefully considering subtle chemical differences between the “war head” group and its intended biological target is vital to design both selective and sensitive chemical probes.

## Data Availability

The original contributions presented in the study are included in the article/Supplementary Material, further inquiries can be directed to the corresponding author.
