# Supplementary material for: Mollugin reacts with phenol thiol but not produce modification on cysteine discovered by a phenol thiol probe
Source: Front Cell Dev Biol. 2025 Aug 20;13:1629762. doi: 10.3389/fcell.2025.1629762 (PMC12405156; doi:10.3389/fcell.2025.1629762)
Supplement: Supplementary file 1 [file DataSheet1.pdf]

Supplementary Material

1. The details of the HPLC gradient and the settings of mass spectrometry 4

Table S1. Method parameters of Chromatography 4

Table S2. Method parameters of mass spectrometry 5

2. The reactions of Probe 1 with *Rubia cordifolia* L. extract or Mollugin 5

3. NMR and HR-MS spectra of compound 5 and Mollugin standard 5

2.1 NMR and HR-MS spectra of compound 5 5

Figure S1. ^{1}H NMR spectrum of compound 5 in DMSO-d_6 6

Figure S2. ^{13}C NMR spectrum of compound 5 in DMSO-d_6 6

Figure S3. HR-MS structural identification of compound 5 7

2.2 NMR and HR-MS spectra of Mollugin standard 7

Figure S4. ^{1}H NMR spectrum of Mollugin in $CDCl_3$ 8

Figure S5. ^{13}C NMR spectrum of Mollugin in $CDCl_3$ 8

Figure S6. HR-MS structural identification of Mollugin 9

4. The structre confirmation of the adduct of Mollugin and Probe-1 with MS^2 9

Figure S7. The structure for fragments in the MS^2 spectrum of the adduct 9

5. The reactions of compound 5 (Mollugin) with cysteine or N-acetyl-L-cysteine 9

6. The reactions of Mollugin with 3,5-dimethyl phenol thiol (DPT) 10

5.1 The methods of the reactions of Mollugin with DPT 10

5.2 NMR and HR-MS of compound 6 10

Figure S8. ^{1}H NMR spectrum of compound 6 in $CDCl_3$ 11

frontiers

Figure S9. ^{13}C NMR spectrum of compound 6 in $CDCl_3$ 11

Figure S10. DEPT 135 spectrum of compound 6 in $CDCl_3$ 12

Figure S11. ^{1}H-^{1}H COSY spectrum of compound 6 in $CDCl_3$ 12

Figure S12. HSQC spectrum of compound 6 in $CDCl_3$ 13

Figure S13. HMBC spectrum of compound 6 in $CDCl_3$ 13

Figure S14. HR-MS structural identification of compound 6 in $CDCl_3$ 14

5.3 Compound 5 (isolated from *Rubia cordifolia* L.) was reacted with DPT and compared with Compound 6 via LC-MS. 14

Figure S15. Chromatogram revealed peaks corresponding to the *m/z* of 437.1430. 15

7. The reactions of Mollugin with various alkyl thiols 15

8. The reactions of Mollugin with various thiol phenols 15

9. Effect of various additives on the reaction of Mollugin and DPT 16

Figure S16. The chromatographic peak corresponding to compound 6 (*m/z*: 437.1430) 16

Figure S17. The chromatographic peak corresponding to the internal standard (*m/z*: 231.1380) ... 17

Figure S18. The chromatographic peak corresponding to the intended product (*m/z*: 499.0584) .. 17

10. TEMPO quenched the reaction of Molugin and DPT 17

Figure S19. ^{1}H NMR spectrum of compound 11 in $CDCl_3$ 18

Figure S20. ^{13}C NMR spectrum of compound 11 in $CDCl_3$ 19

Figure S21. HR-MS structural identification of compound 11 19

11. Synthesis of related compounds 20

11.1 Experimental Part 20

11.2 NMR and HR-MS data 22

Figure S22. ^{1}H NMR spectrum of compound 7 in $CDCl_3$ 22

Figure S23. ^{13}C NMR spectrum of compound 7 in $CDCl_3$ 22

Figure S24. HR-MS spectrum of compound 7 23

Figure S25. ^{1}H NMR spectrum of compound 8 in $CDCl_3$ 23

Figure S26. ^{13}C NMR spectrum of compound 8 in $CDCl_3$ 24

Figure S27. HR-MS spectrum of compound 8 24

Figure S28. ^{1}H NMR spectrum of compound 13 in CD_3OD 25

Figure S29. ^{13}C NMR spectrum of compound 13 in CD_3OD 25

Figure S30. HR-MS spectrum of compound 13 26

Figure S31. ^{1}H NMR spectrum of compound 14 in CD_3OD 26

Figure S32. ^{13}C NMR spectrum of compound 14 in CD_3OD 27

Figure S33. HR-MS spectrum of compound 14 27

General Methods and Information of Chemistry

All solvents were commercially available and used without further purification unless noted. The chemicals were purchased from commercial sources. Thin-layer chromatography (TLC) was performed using silica gel plates (HSGF254, 0.4–0.5 mm) purchased from Yantai Jiangyou Silica Gel Development Co., Ltd.. Visualization of the developed chromatogram was performed by UV absorbance (254 or 365 nm), dinitrophenylhydrazine, ceric ammonium molybdate, potassium permanganate or anisaldehyde stain. Column chromatography was performed using EcoChrom ICN SiliTech 32-63 D silica gel (300−400 mesh) using the indicated solvent system as eluent. The ^{1}H and ^{13}C nuclear magnetic resonance (NMR) spectra were recorded on a model 400 Bruker Avance spectrometer or a model 600 Bruker Avance spectrometer. An Agilent ProStar 218 HPLC system equipped with an C18 column was used to purify the key products.

1. The details of the HPLC gradient and the settings of mass spectrometry

The details of the HPLC gradient and the settings of mass spectrometry were described as follows:

Table S1. Method parameters of Chromatography (Dionex UltiMate 3000, Hypersil GOLD-C18) ：

Solvents：A：H_2O（0.1% FA）； B：MeCN；

Flow rate：0.3 mL/min，Injection volume：2 μL

Multi-Step Gradient:

Time(min)	A%	B%
0	70	30
1	70	30
5	40	60
16	5	95
17	5	95
17.1	70	30
20	70	30

Table S2. Method parameters of mass spectrometry (Thermofisher QE)

General	
Runtime	0 to 20 min
In-source CID	0.0 eV
Polarity	Positive
FULL MS -SIM	
Resolution	70,000
AGC target	1e6
Maximum IT	100 ms
Scan range	100 to 800 *m/z*
PRM-MS^2	
Resolution	17,500
AGC target	1e5
Maximum IT	50 ms
Isolation window	0.8 *m/z*
Isolation offset	0.0 *m/z*
(N)CE/ stepped (N)CE	Ce: 30

2. The reactions of Probe 1 with *Rubia cordifolia* L. extract or Mollugin

Rubia cordifolia L. extract (5mg) or Mollugin (2mg), **Probe 1** (2mg) and ascorbic acid (3mg) were mixed and dissolved in 1ml ultra-dry anhydrous methanol (reaction solvent should be deaerated in advance, filled with argon). After the mixture has been stirred at 25°C for 22 hours, stop the reaction. The reaction solvent was removed with vacuum and the residue was stored in -40°C as working samples for next analysis. Right before the analysis by HPLC-MS^n, the working sample was dissolved with methanol to afford a 30 μg/mL (30 ppm) solution in the total weight of *Rubia cordifolia* L. extract or Mollugin, **Probe 1** and ascorbic acid.

3. NMR and HR-MS spectra of compound 5 and Mollugin standard

2.1 NMR and HR-MS spectra of compound 5

^{1}H NMR (500 MHz, DMSO-d_6) δ 11.22 (s, 1H), 8.21 (d, J = 8.4 Hz, 1H), 8.06 (d, J = 8.3 Hz, 1H), 7.64 (t, J = 7.5 Hz, 1H), 7.56 (t, J = 7.6 Hz, 1H), 6.87 (d, J = 10.0 Hz, 1H), 5.80 (d, J = 10.0 Hz, 1H), 3.93 (s, 3H), 1.42 (s, 6H). **HR-MS** *m/z* $[M-H]^-$ calcd for $C_{17}H_{15}O_4$ 283.0976; found 283.0977.

^{13}C NMR (126 MHz, DMSO-d_6) δ 170.58, 151.84, 141.21, 130.36, 129.42, 128.22, 127.83, 127.01, 125.14, 123.84, 121.96, 121.57, 112.87, 106.21, 75.41, 53.06, 27.05.

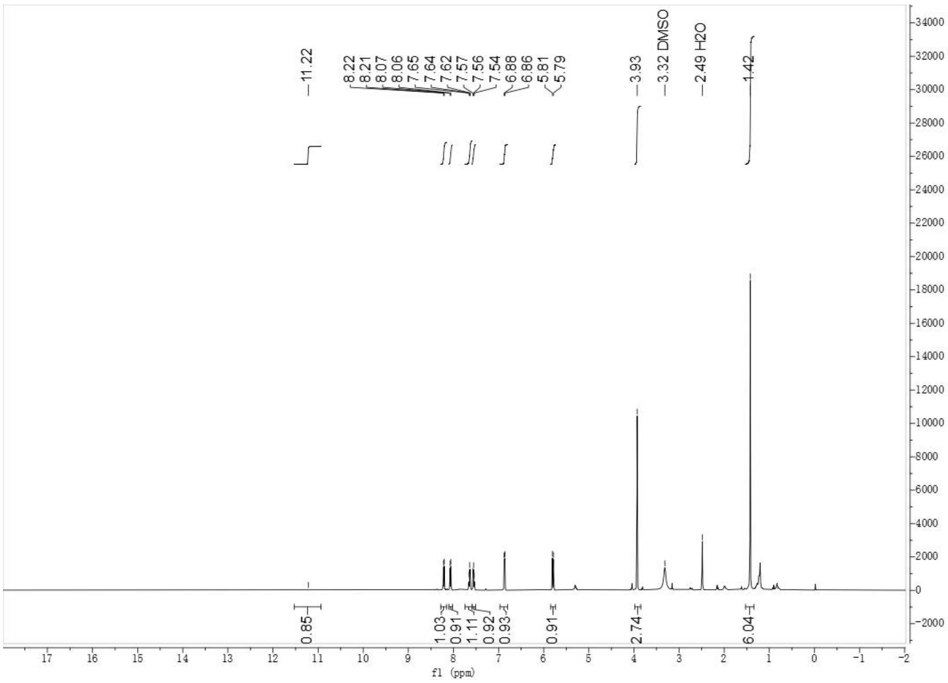

Figure S1. ^{1}H NMR spectrum of compound 5 in DMSO-*d*₆

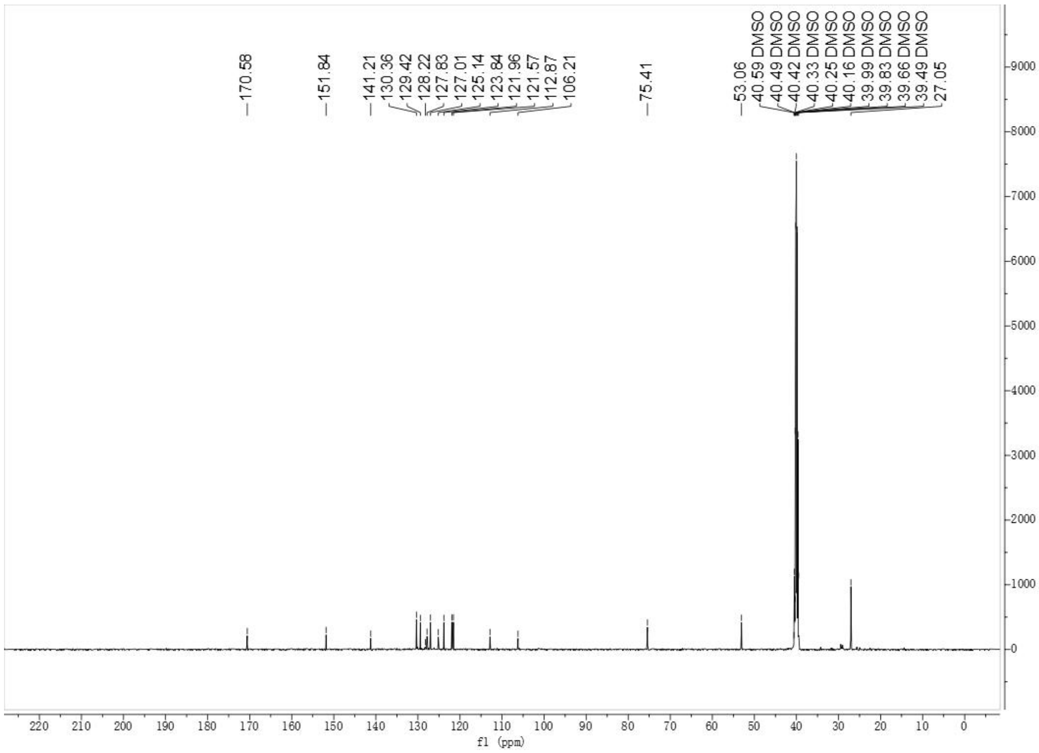

Figure S2. ^{13}C NMR spectrum of compound 5 in DMSO-*d*₆

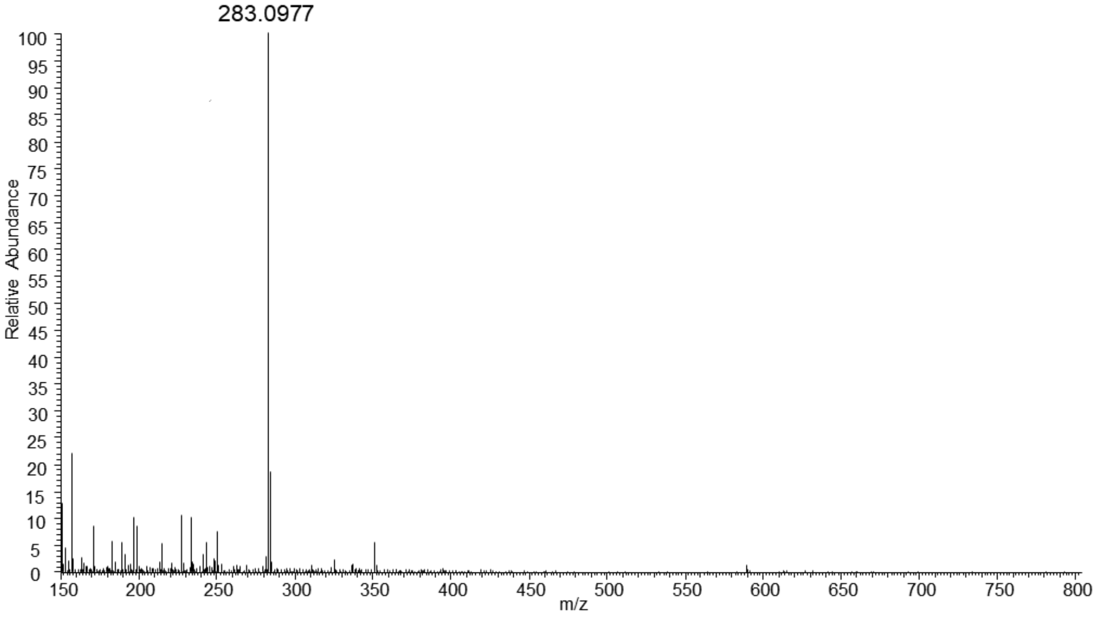

Figure S3. HR-MS structural identification of compound 5

2.2 NMR and HR-MS spectra of Mollugin standard

^{1}H NMR (400 MHz, $CDCl_3$) δ 12.18 (s, 1H), 8.39 (ddd, *J* = 8.3, 1.3, 0.7 Hz, 1H), 8.23 – 8.16 (m, 1H), 7.63 (ddd, *J* = 8.3, 6.9, 1.3 Hz, 1H), 7.53 (ddd, *J* = 8.2, 6.9, 1.3 Hz, 1H), 7.13 (d, *J* = 10.0 Hz, 1H), 5.69 (d, *J* = 10.0 Hz, 1H), 4.04 (s, 3H), 1.52 (s, 6H). **HR-MS** *m/z* $[M-H]^-$ calcd for $C_{17}H_{15}O_4$ 283.0976; found 283.0978.

^{13}C NMR (101 MHz, CDCl3) δ 172.49, 156.49, 141.57, 129.33, 129.01, 128.85, 126.29, 125.08, 124.02, 122.31, 121.92, 112.56, 102.22, 77.23, 74.65, 52.29, 26.86.

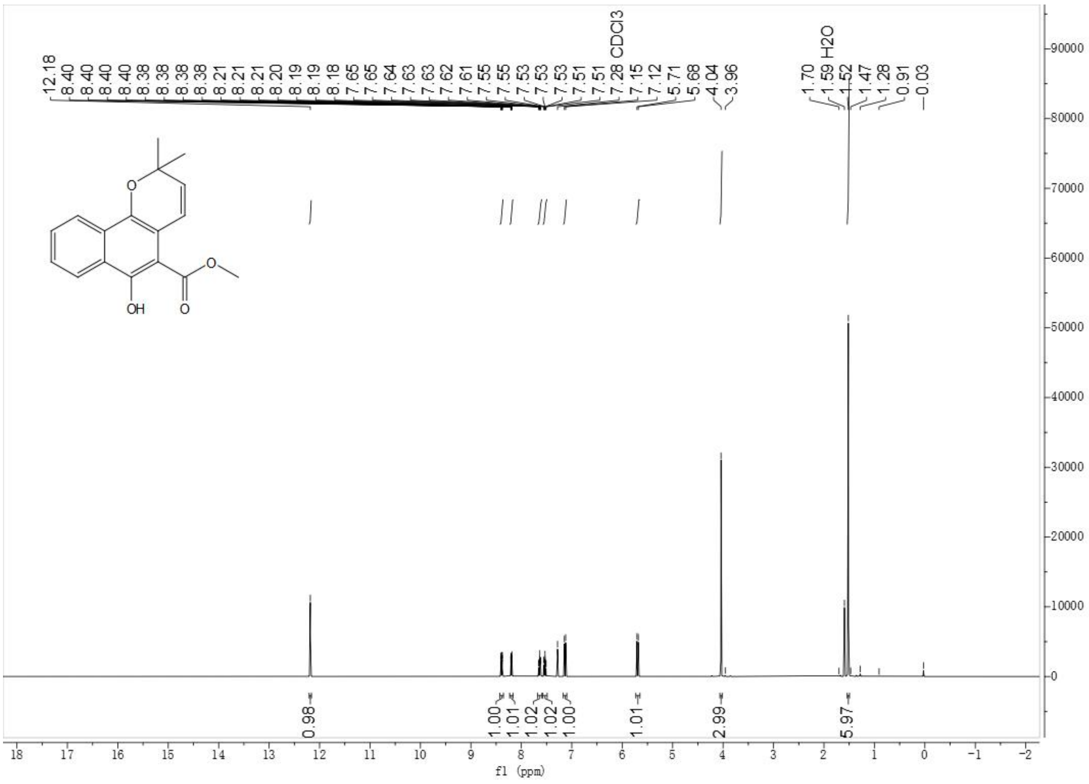

Figure S4. ^{1}H NMR spectrum of Mollugin in $CDCl_3$

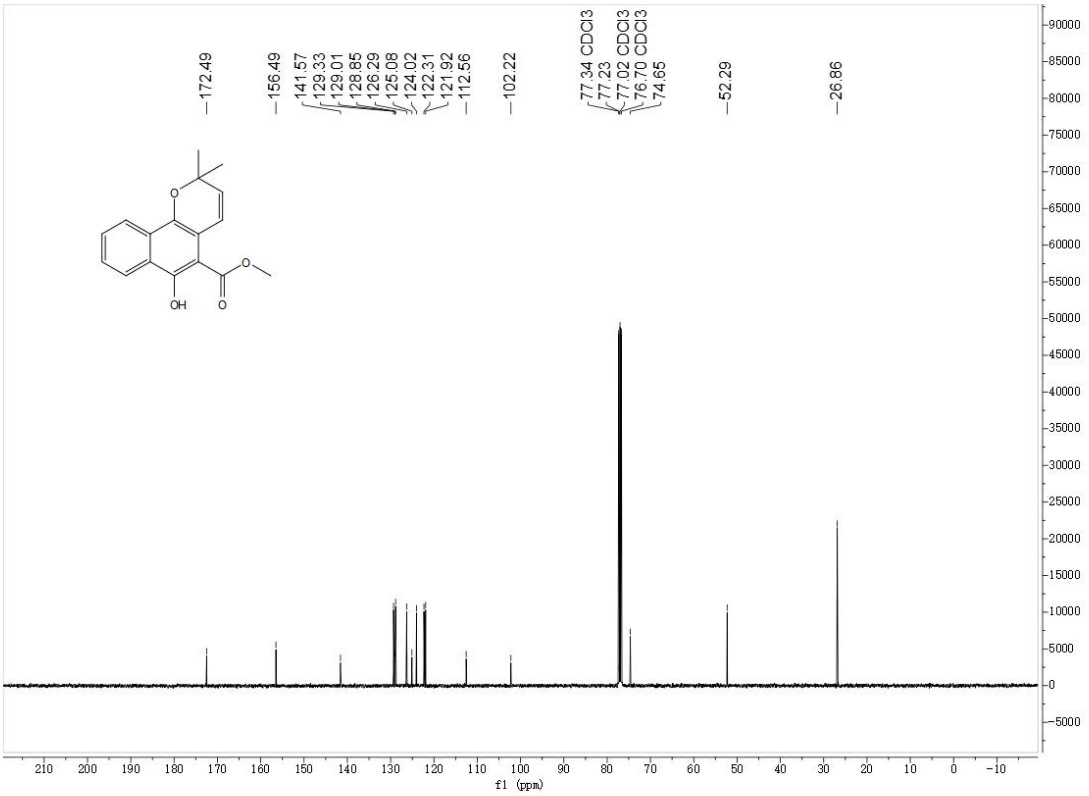

Figure S5. ^{13}C NMR spectrum of Mollugin in $CDCl_3$

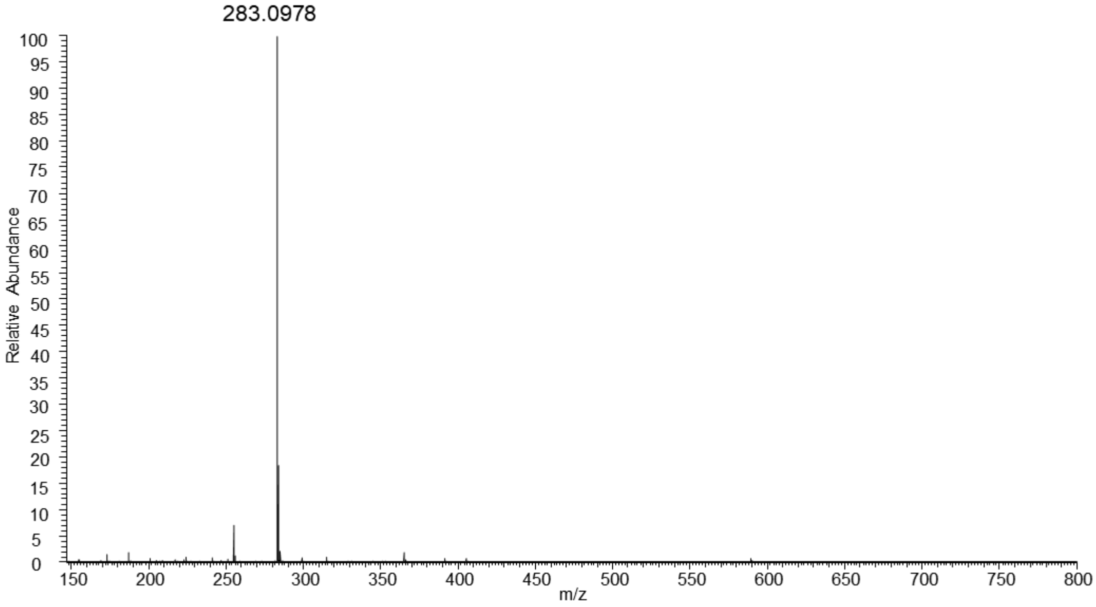

Figure S6. HR-MS structural identification of Mollugin

4. The structre confirmation of the adduct of Mollugin and Probe-1 with MS^2

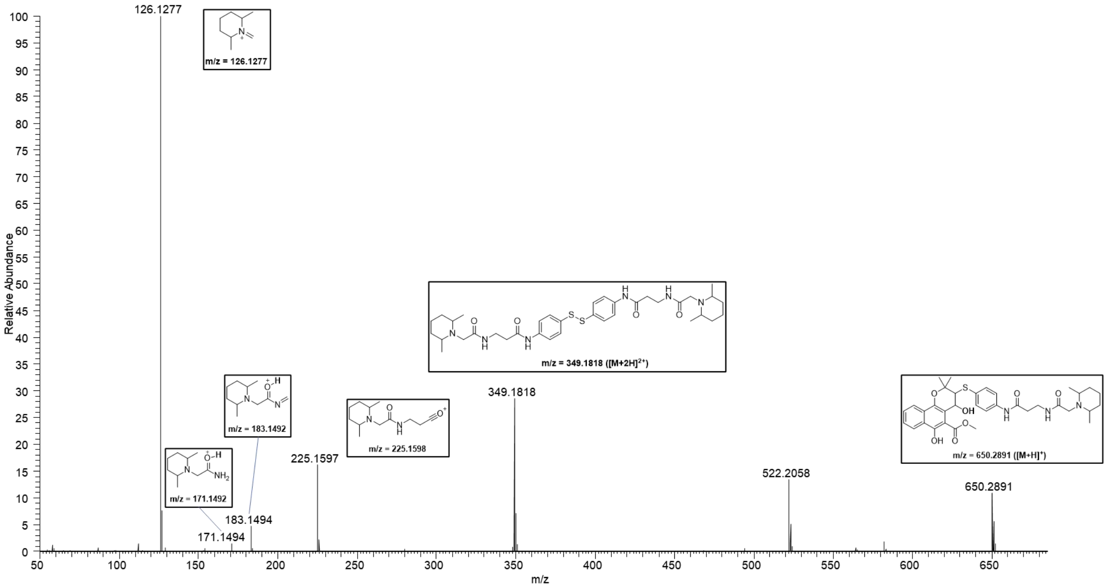

Figure S7. The structure for fragments in the MS^2 spectrum of the adduct

5. The reactions of compound 5 (Mollugin) with cysteine or N-acetyl-L-cysteine

Mollugin (10 mg, 0.035 mmol, 1.0 eq), cysteine (5 mg, 0.042 mmol, 1.2 eq) were dissolved in DMSO (2 mL) in a 4 mL flask. Then the solution was stirred in 25℃ and the reaction was monitored by TLC. No products had been monitored within 24 hours.

Mollugin (10 mg, 0.035 mmol, 1.0 eq), N-acetyl-L-cysteine (7 mg, 0.042 mmol, 1.2 eq) were dissolved in DMSO (2 mL) in a 4 mL flask. Then the solution was stirred in 25℃ and the reaction was monitored by TLC. No products had been monitored within 24 hours. The experiment was repeated with ethanol or THF instead of DMSO, and no product was found within 24h still.

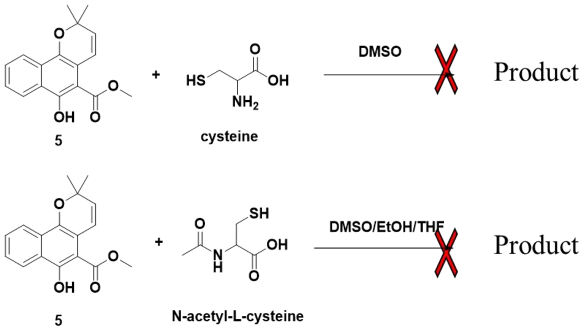

6. The reactions of Mollugin with 3,5-dimethyl phenol thiol (DPT)

5.1 The methods of the reactions of Mollugin with DPT

Mollugin (100 mg, 0.35 mmol, 1.0 eq), DPT (55 μL, 0.42 mmol, 1.2 eq) were dissolved in CH_2Cl_2 (20 mL) in a 50 mL flask. The reaction was stirred in 25℃ for 2 hours. The solvent then was removed with rotary evaporator and the residue was purified with silica gel chromatography (Hexane/Ethyl acetate, 100:4) to afford orange solid product **6** (32 mg). **HR-MS** *m/z* $[M-H]^-$ calcd for $C_{25}H_{25}O_5S$ 437.1428; found 437.1430. Yield=20.8%

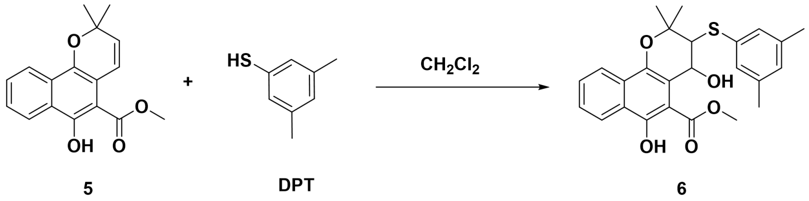

5.2 NMR and HR-MS of compound 6

^{1}H NMR (400 MHz, $CDCl_3$) δ 11.19 (s, 1H), 8.42 – 8.36 (m, 1H), 8.21 – 8.15 (m, 1H), 7.67 – 7.62 (m, 1H), 7.59 (dd, *J* = 8.2, 1.3 Hz, 1H), 7.18 (s, 2H), 6.89 (s, 1H), 5.26 (d, *J* = 8.4 Hz, 1H), 4.04 (s, 3H), 3.50 (d, *J* = 8.5 Hz, 1H), 3.31 (s, 1H), 2.30 (s, 6H), 1.65 (s, 3H), 1.53 (s, 3H). **HR-MS** *m/z* $[M-H]^-$ calcd for $C_{25}H_{25}O_5S$ 437.1428; found 437.1430.

^{13}C NMR (101 MHz, $CDCl_3$) δ 171.58, 155.14, 141.27, 138.82, 135.31, 129.25, 129.04, 128.90, 128.66, 126.89, 125.62, 123.84, 122.34, 112.92, 104.62, 79.00, 68.27, 61.02, 52.59, 27.91, 21.19, 20.72.

frontiers

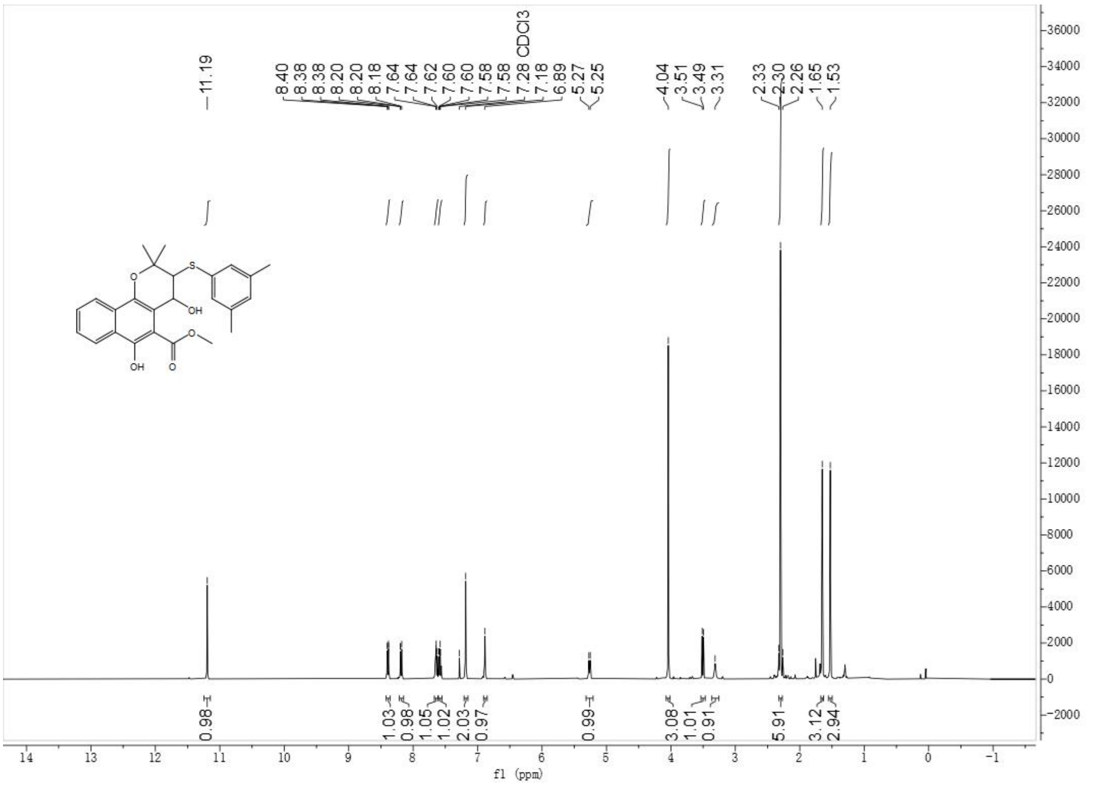

Figure S8. ^{1}H NMR spectrum of compound 6 in $CDCl_3$

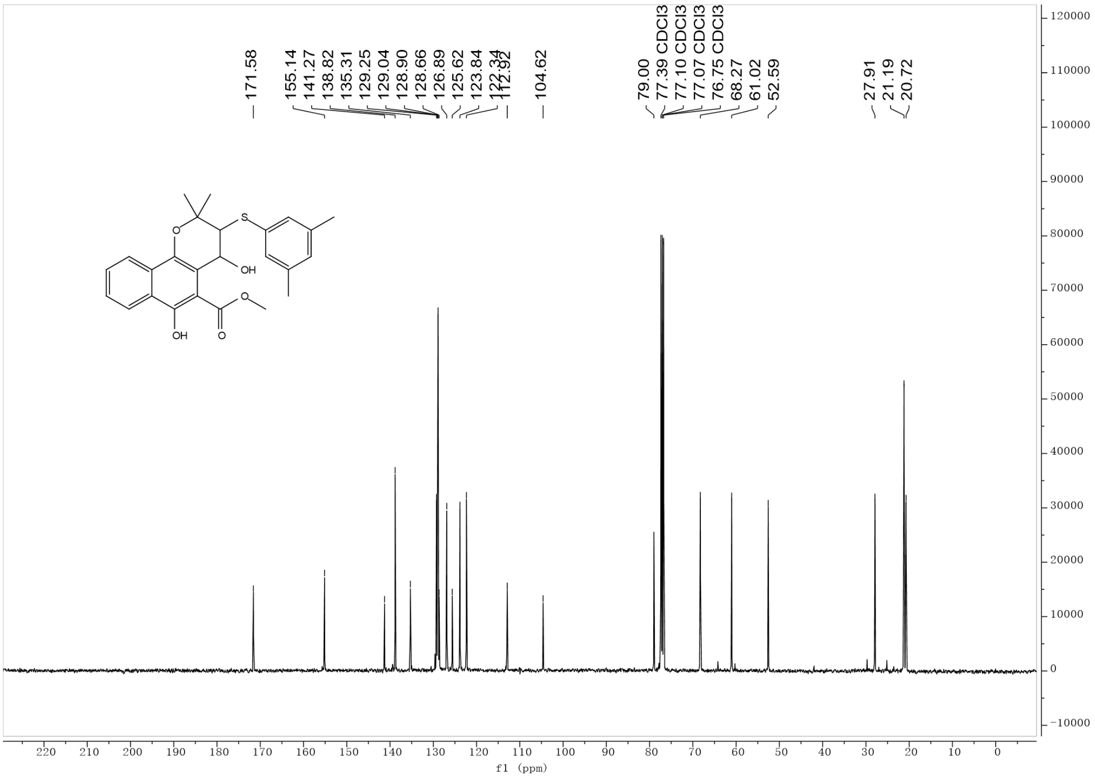

Figure S9. ^{13}C NMR spectrum of compound 6 in $CDCl_3$

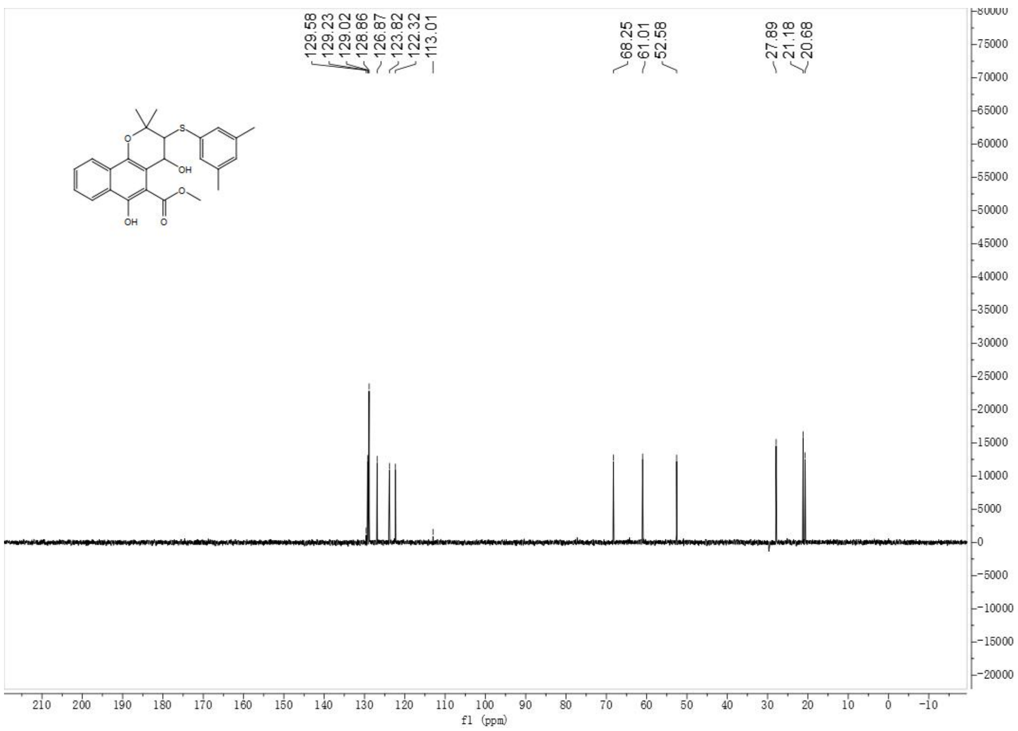

Figure S10. DEPT 135 spectrum of compound 6 in $CDCl_3$

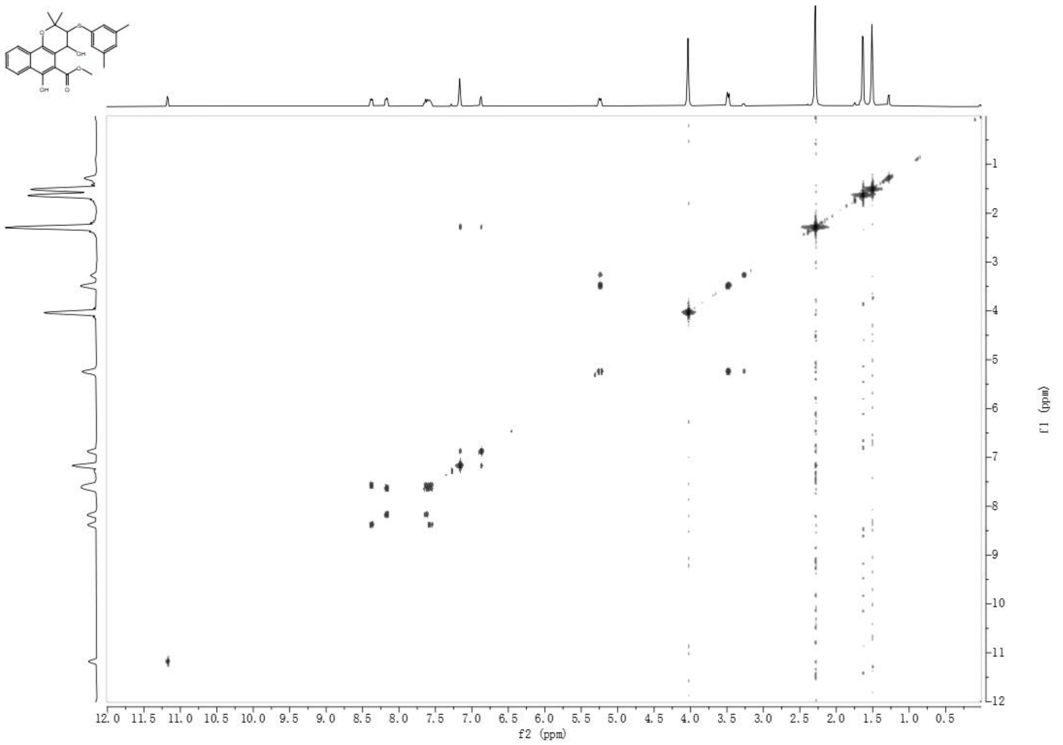

Figure S11. ^1H-^1H COSY spectrum of compound 6 in $CDCl_3$

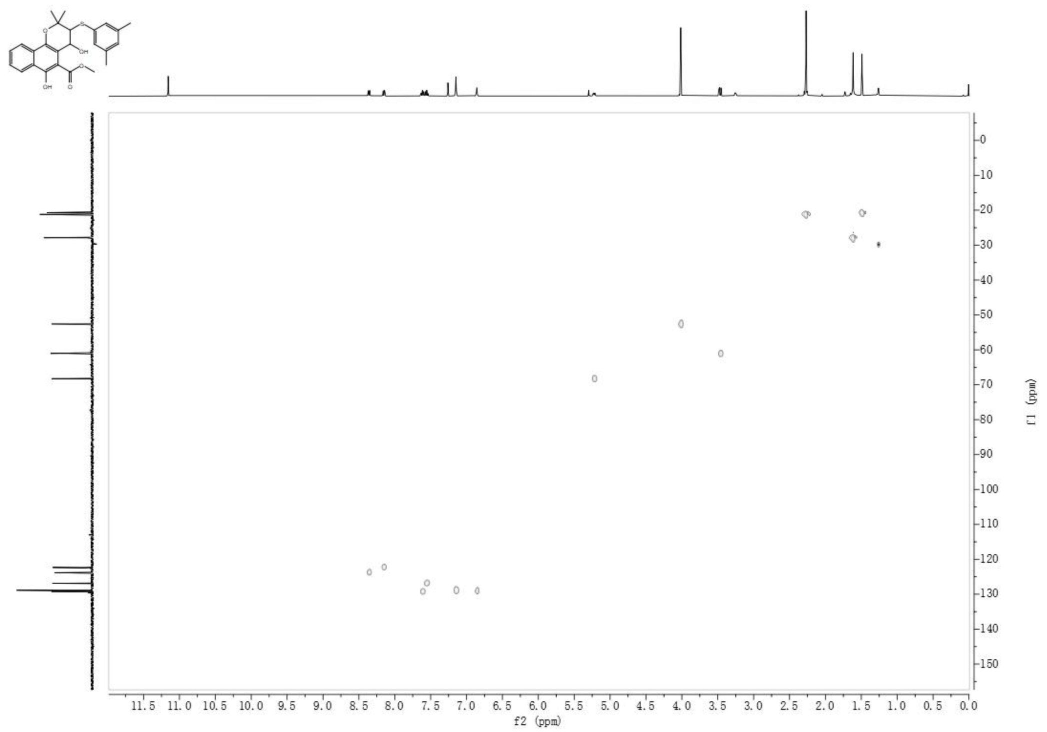

Figure S12. HSQC spectrum of compound 6 in $CDCl_3$

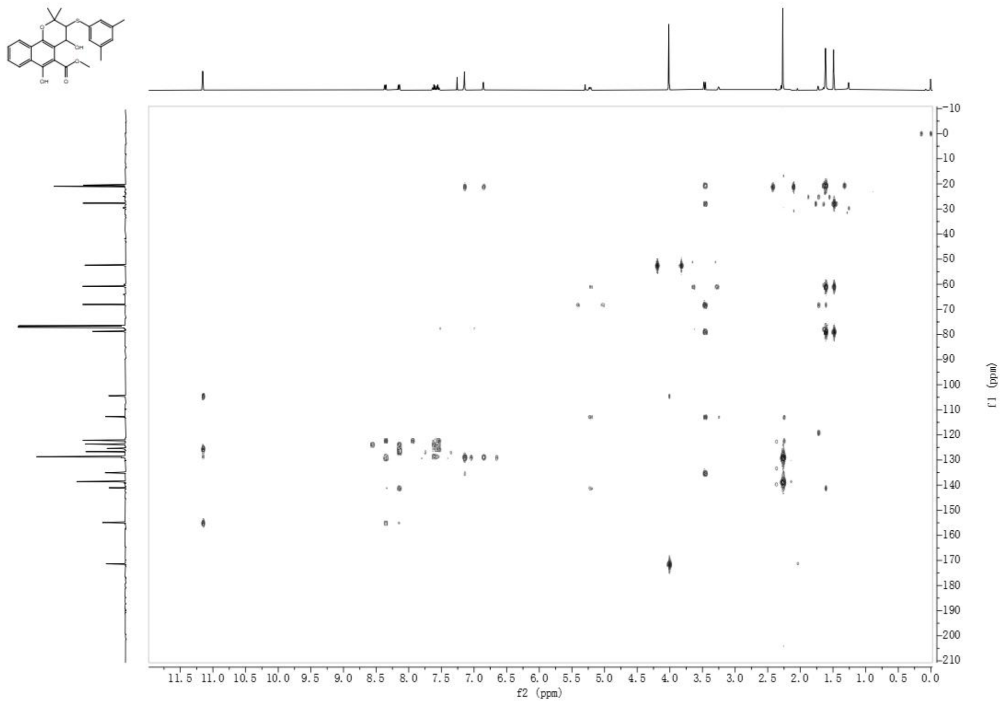

Figure S13. HMBC spectrum of compound 6 in $CDCl_3$

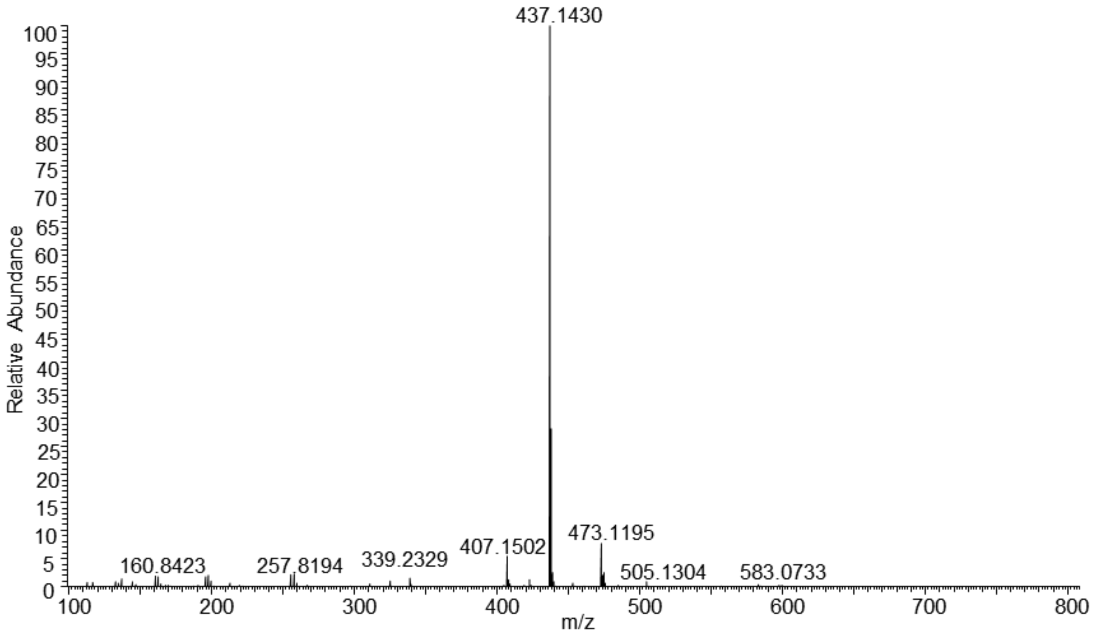

Figure S14. HR-MS structural identification of compound 6 in $CDCl_3$

5.3 Compound 5 (isolated from *Rubia cordifolia* L.) was reacted with DPT and compared with Compound 6 via LC-MS.

Compound **6** (3mg) was diluted to 10 ppm with DMF to obtain the test solution "I". The isolate (isolated from *Rubia cordifolia* L.) (3 mg) and DPT (2 μL) were dissolved in DMF (1 mL), stirred at room temperature for 24 h, and the reaction solution was diluted to 50 ppm at the end of the reaction to obtain the test solution "II" . 5 μL of "I" and 45 μL of "II" were mixed to obtain "III". All the liquids to be tested were tested by HPLC-MS, and the chromatographic peak with molecular weight of 437.1430 was selected.

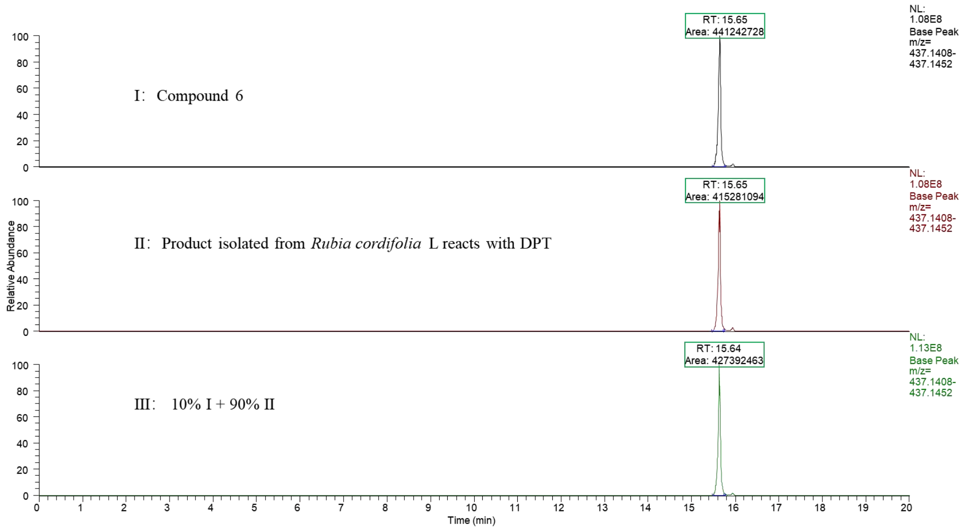

Figure S15. Chromatogram revealed peaks corresponding to the *m/z* of 437.1430.

7. The reactions of Mollugin with various alkyl thiols

Mollugin (**5**) (10 mg, 0.035 mmol, 1.0 eq), various alkyl thiols (**6a**-**9a**) (4-6 μL, 0.042 mmol, 1.2 eq) were dissolved in CH_2Cl_2 (2 mL) in a 4 mL flask. Then the solution was stirred in 25°C and the reaction was monitored by TLC. No products were detected in all reactions within 24 hours.

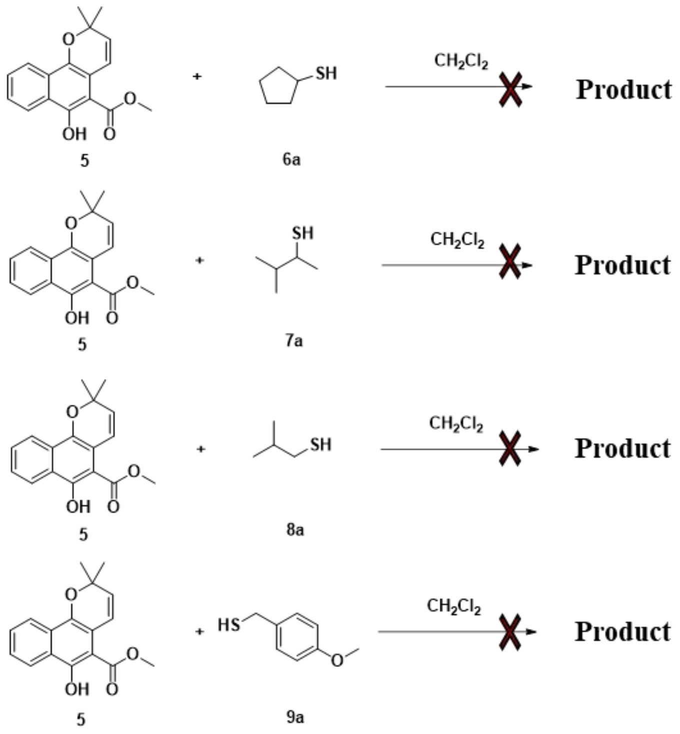

8. The reactions of Mollugin with various thiol phenols

Mollugin (**5**) (10 mg, 0.035 mmol, 1.0 eq), various thiol phenols (**1a**-**4a**) (4-6 μl, 0.042 mmol, 1.2 eq) were dissolved in CH_2Cl_2 (2 mL) in a 4 mL flask. Then the solution was stirred in 25°C and the reaction was monitored by TLC. The corresponding products (**7-10**) could be detected successively in each reaction within half an hour to 2 hours, and all reactions were completed within 4 hours. Then these reactions solvent were removed with vacuum and the residue were stored in -40°C as analysis samples. Right before the analysis by LC-MS, these samples were dissolved with methanol to afford a 20 μg/mL (20 ppm) solution in the total weight.

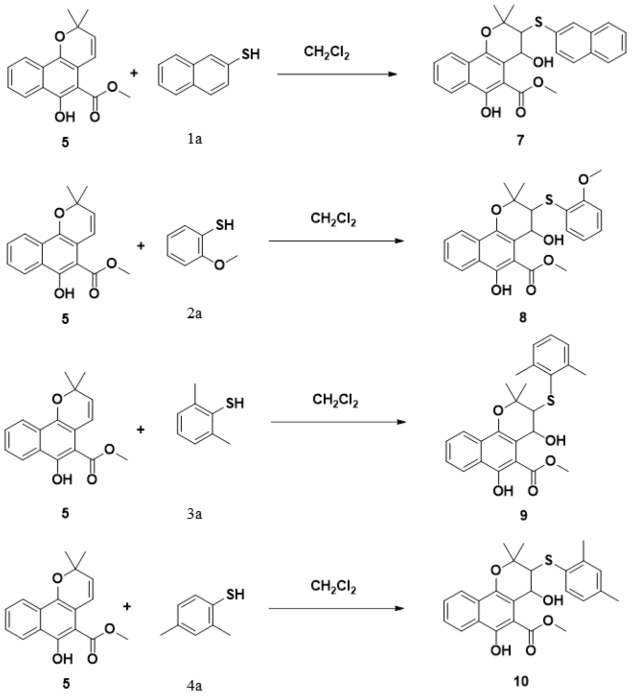

9. Effect of various additives on the reaction of Mollugin and DPT

First, take four 4 mL reaction vials and label them sequentially as ①-④. Add 2 μL of DPT solution dissolved in 0.8 mL DMF to each vial. Subsequently, add 5 mg of TEMPO to vial ②, 5 μL of TBHP to vial ③, and 5 mg of NaBr to vial ④. Stir all four groups of samples simultaneously for 10 minutes. Then, add 3 mg of Mollugin to each reaction vial and continue stirring overnight. After the reaction, add 10 μL of 0.75 mg/mL dehydrocostus lactone solution as an internal standard to each vial and mix thoroughly. Finally, dilute all reaction solutions to a concentration of 30 ppm and perform HPLC-MS analysis separately.

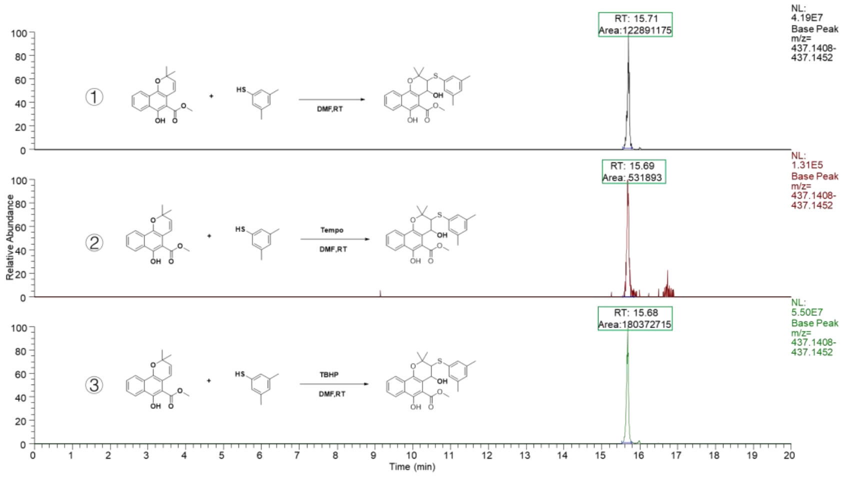

Figure S16. The chromatographic peak corresponding to compound 6 (*m/z*: 437.1430)

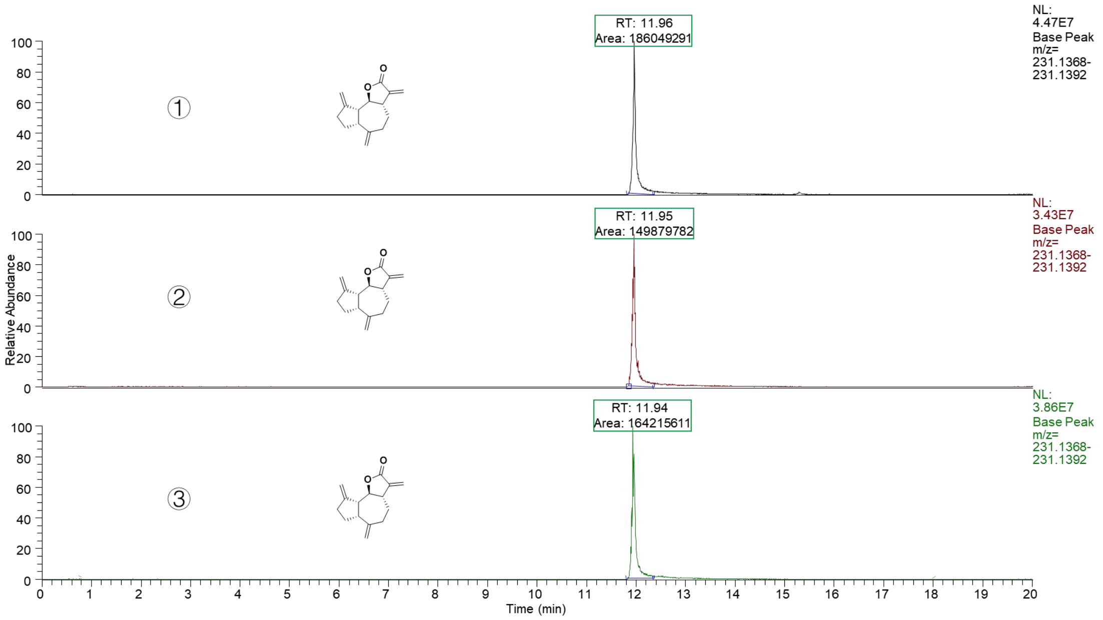

Figure S17. The chromatographic peak corresponding to the internal standard (*m/z*: 231.1380)

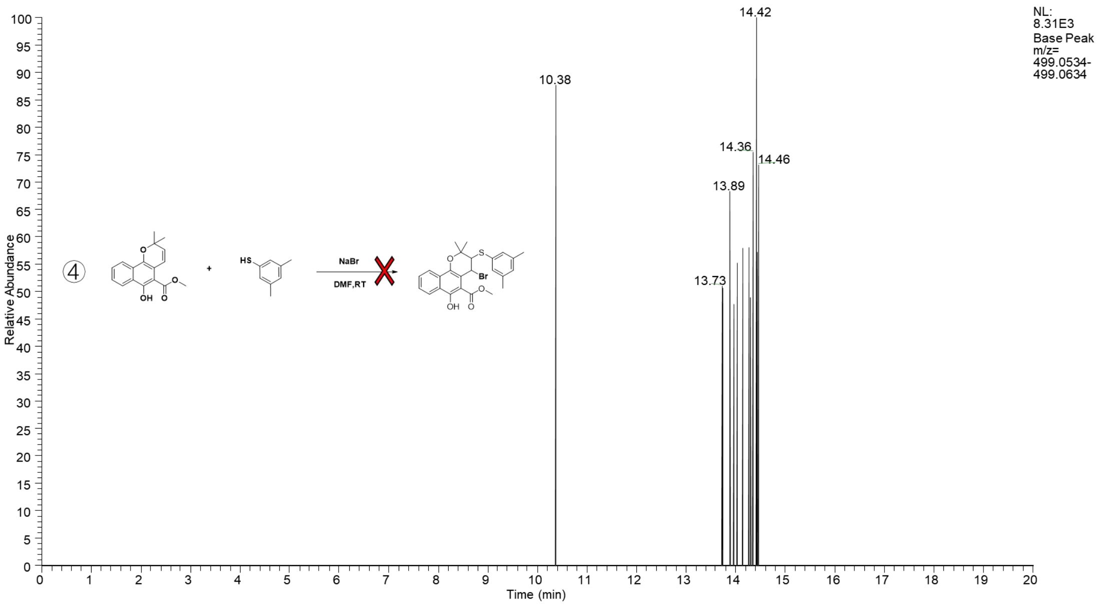

Figure S18. The chromatographic peak corresponding to the intended product (*m/z*: 499.0584)

10. TEMPO quenched the reaction of Molugin and DPT

DPT (55 µL, 0.42mmol, 1.2 eq) and TEMPO (109.4mg, 0.7mmol, 2 eq) were dissolved in DMF (4mL) in a 50 mL flask and stirred for 5min. Mollugin (100 mg, 0.35 mmol, 1.0 eq) was then added to the above solution and continued stirring. The

reaction process was monitored by TLC, and no detectable formation of compound **6** was observed throughout the 12 h monitoring period. Then the reaction was stopped and the product was separated and purified by silica gel column to obtain compound **11** (25 mg). **^{1}H NMR** (500 MHz, $CDCl_3$) δ 7.27 (d, *J* = 1.5 Hz, 2H), 7.00 (s, 1H), 2.37 (s, 6H), 1.85 (s, 1H), 1.66 (s, 5H), 1.57 (s, 4H), 1.50 (s, 4H), 1.41 – 1.32 (m, 1H), 1.27 (d, *J* = 8.2 Hz, 1H), 0.95 (s, 3H).**^{13}C NMR** (126 MHz, $CDCl_3$) δ 150.02, 138.28, 131.02, 123.54, 61.25, 58.74, 43.55, 41.38, 35.39, 32.65, 28.75, 27.96, 21.40, 17.31. **HR-MS** *m/z* $[M+H]^+$ calcd for $C_{17}H_{28}NOS$ 294.1886; found 294.1887.

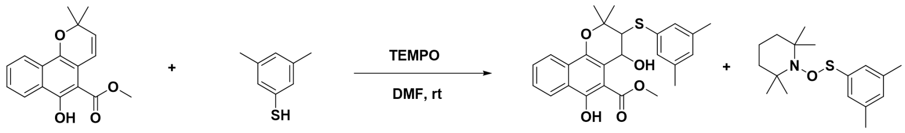

5 DPT 6 11

0%

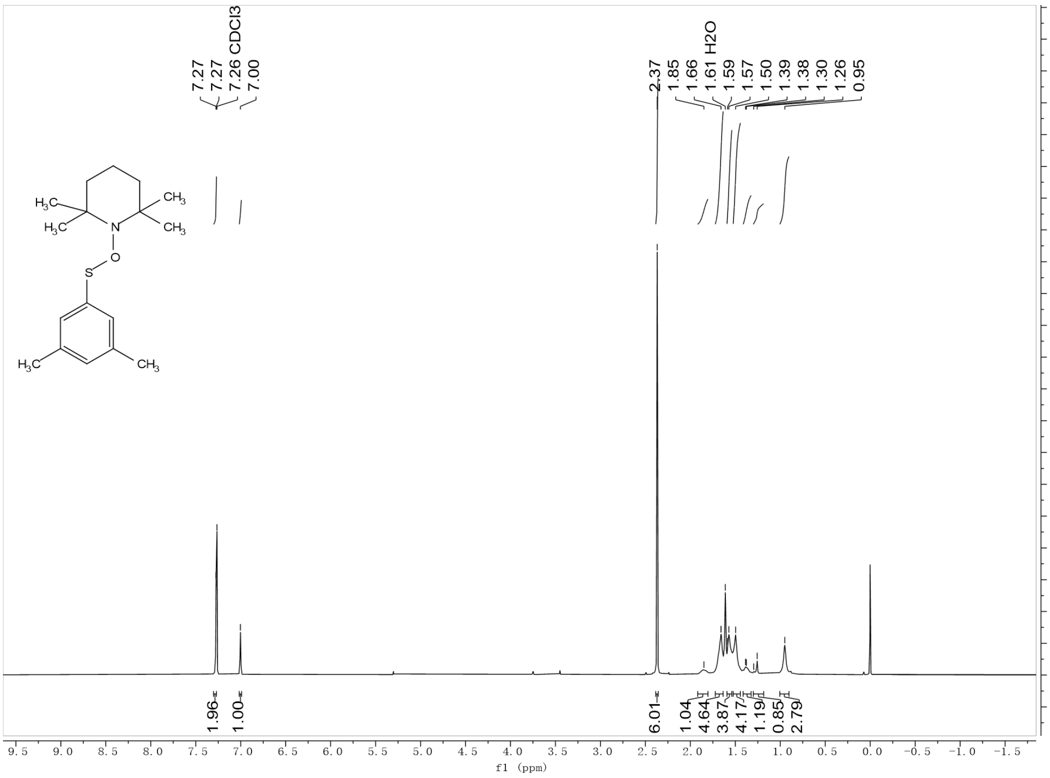

Figure S19. ^{1}H NMR spectrum of compound 11 in $CDCl_3$

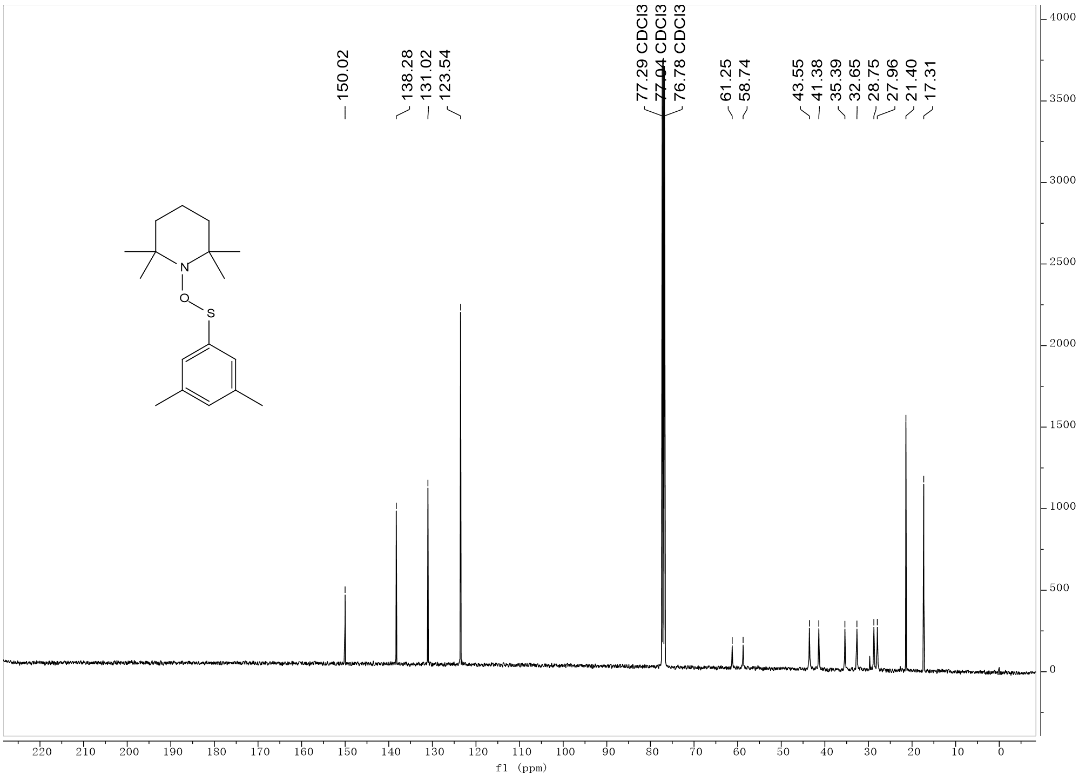

Figure S20. ^{13}C NMR spectrum of compound 11 in $CDCl_3$

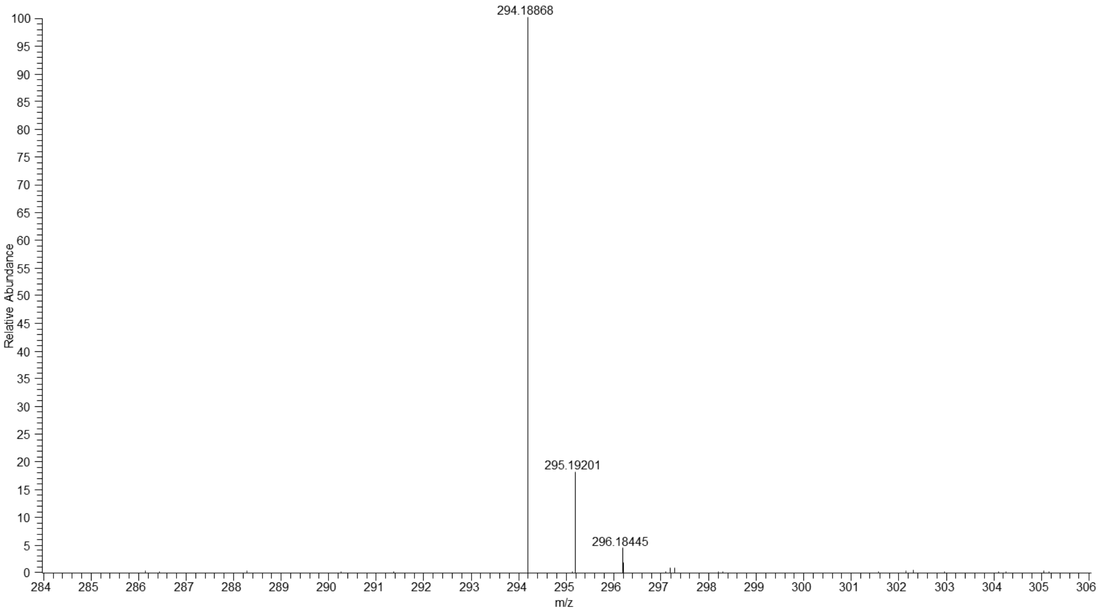

Figure S21. HR-MS structural identification of compound 11

11. Synthesis of related compounds

11.1 Experimental Part

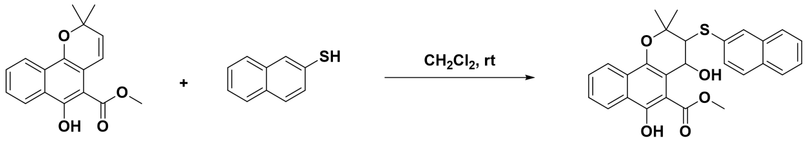

5 1a 7

Mollugin (**5**) (50 mg, 0.18 mmol, 1.0 eq), 2-Naphthalenethiol (**1a**) (32 mg, 0.21 mmol, 1.2 eq) were dissolved in CH_2Cl_2 (4 mL) in a 25 mL flask. The reaction was stirred in 25°C for 30 min. The solvent then was removed with rotary evaporator and the residue was purified with silica gel chromatography (Hexane/Ethyl acetate, 100:3) to afford yellow oily product **7** (10.83 mg). **HR-MS** *m/z* $[M-H]^-$ calcd for $C_{27}H_{23}O_5S$ 459.1272; found 459.1276. Yield=13.5%. **^{1}H NMR** (500 MHz, CDCl3) δ 11.23 (s, 1H), 8.39 – 8.35 (m, 1H), 8.18 – 8.13 (m, 1H), 8.01 (d, *J* = 1.9 Hz, 1H), 7.82 – 7.74 (m, 3H), 7.64 – 7.59 (m, 2H), 7.57 (ddd, *J* = 8.3, 6.9, 1.4 Hz, 1H), 7.50 – 7.44 (m, 2H), 5.29 (dd, *J* = 8.3, 4.1 Hz, 1H), 4.00 (s, 3H), 3.63 (d, *J* = 8.2 Hz, 1H), 3.33 (d, *J* = 4.5 Hz, 1H), 1.63 (s, 3H), 1.54 (s, 3H). **^{13}C NMR** (126 MHz, CDCl3) δ 171.49, 155.46, 141.25, 133.72, 133.26, 132.24, 129.70, 129.35, 128.83, 128.77, 127.73, 127.32, 126.95, 126.72, 126.24, 125.64, 123.87, 122.37, 112.92, 104.43, 78.89, 68.21, 60.92, 52.66, 27.85, 20.99.

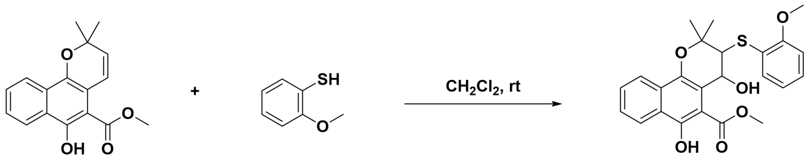

5 2a 8

Mollugin (**5**) (50 mg, 0.18 mmol, 1.0 eq), 2-Methoxythiophenol (**2a**) (30 mg, 0.21 mmol, 1.2 eq) were dissolved in CH_2Cl_2 (3 mL) in a 10 mL flask. The reaction was stirred in 25°C for 4 h. The solvent then was removed with rotary evaporator and the residue was purified with silica gel chromatography (Hexane/Ethyl acetate, 100:3) to afford gray solid product **8** (15.81 mg). **HR-MS** *m/z* $[M+Na]^+$ calcd for $C_{24}H_{24}O_6SNa$ 463.1186; found 463.1187. Yield=19.0%.**^{1}H NMR** (500 MHz, $CDCl_3$) δ 10.95 (s, 1H), 8.34 (dd, *J* = 8.3, 1.4 Hz, 1H), 8.12 (dt, *J* = 8.4, 0.9 Hz, 1H), 7.59 (ddd, *J* = 8.1, 5.9, 1.6 Hz, 2H), 7.53 (ddd, *J* = 8.2, 6.8, 1.4 Hz, 1H), 7.32 (td, *J* = 7.8, 1.7 Hz, 1H), 6.95 (td, *J* = 7.6, 1.2 Hz, 1H), 6.90 (dd, *J* = 8.4, 1.2 Hz, 1H), 5.21 (dd, *J* = 9.0, 2.2 Hz, 1H), 4.02 (s, 3H), 3.90 (s, 3H), 3.86 (d, *J* = 2.7 Hz, 1H), 3.27 (d, *J* = 9.1 Hz, 1H), 1.62 (s, 3H), 1.49 (s, 3H). **^{13}C NMR** (126 MHz, $CDCl_3$) δ 171.84, 158.70, 154.34, 140.93, 135.28, 130.08, 128.95, 128.44, 126.70, 125.50, 123.71, 122.56, 122.24, 121.33, 113.25, 111.17, 105.12, 79.27, 68.15, 60.55, 55.96, 52.46, 28.06, 19.90.

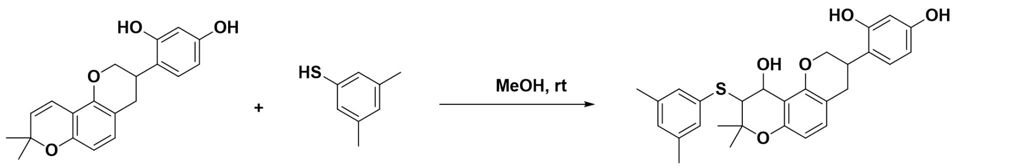

Glabridin (**12**) (100 mg, 0.31 mmol, 1.0 eq), DPT (50 μL, 0.37 mmol, 1.2 eq) were dissolved in MeOH (3 mL) in a 10 mL flask. The reaction was stirred in 25°C for 6 h. The solvent then was removed with rotary evaporator and the residue was purified with silica gel chromatography (Hexane/acetone, 100:10) to afford gray solid product **13** (33.21 mg). **HR-MS** *m/z* [M-H]$^-$ calcd for $C_{28}H_{29}O_5S$ 477.1741; found 477.1746. Yield=22.4%. **^{1}H NMR** (500 MHz, CD_3OD) δ 7.11 (d, *J* = 11.1 Hz, 2H), 6.95 – 6.84 (m, 3H), 6.34 – 6.29 (m, 2H), 6.27 (dt, *J* = 8.3, 2.2 Hz, 1H), 4.90 (d, *J* = 5.0 Hz, 1H), 4.35 (dddd, *J* = 21.2, 10.3, 3.6, 2.1 Hz, 1H), 4.01 (dt, *J* = 35.8, 10.3 Hz, 1H), 3.49 (ddd, *J* = 12.3, 9.6, 4.3 Hz, 1H), 3.45 – 3.41 (m, 1H), 2.99 (ddd, *J* = 16.2, 11.2, 5.1 Hz, 1H), 2.79 (dddd, *J* = 15.7, 7.7, 5.2, 2.0 Hz, 1H), 2.27 (d, *J* = 4.4 Hz, 6H), 1.48 (d, *J* = 4.4 Hz, 6H). **^{13}C NMR** (126 MHz, MeOD) δ 156.83, 156.09, 153.55, 151.63, 138.65, 135.63, 129.80, 128.61, 128.34, 127.61, 118.70, 114.68, 111.10, 108.96, 106.50, 102.39, 77.45, 70.22, 65.60, 58.36, 31.64, 30.42, 24.43, 24.13, 20.07.

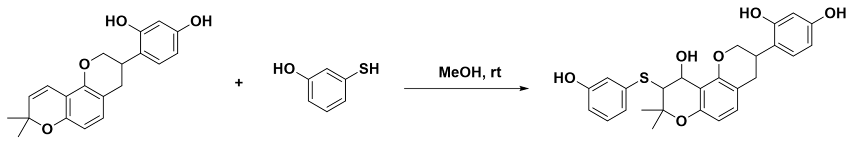

Glabridin (**12**) (100 mg, 0.31 mmol, 1.0 eq), 3-hydroxythiophenol (**10a**) (48 mg, 0.37 mmol, 1.2 eq) were dissolved in MeOH (3 mL) in a 10 mL flask. The reaction was stirred in 25°C for 1.5 h. The solvent then was removed with rotary evaporator and the residue was purified with silica gel chromatography (Hexane/acetone, 100:10) to afford gray solid product **14** (77.2 mg). **HR-MS** *m/z* [M-H]$^-$ calcd for $C_{26}H_{25}O_6S$ 465.1377; found 465.1383. Yield=53.6%. **^{1}H NMR** (500 MHz, CD_3OD) δ 7.12 (tdd, *J* = 7.7, 5.8, 1.6 Hz, 1H), 7.02 – 6.86 (m, 4H), 6.65 (dd, *J* = 7.3, 3.3 Hz, 1H), 6.32 (dq, *J* = 4.7, 2.3 Hz, 2H), 6.27 (dt, *J* = 8.3, 2.1 Hz, 1H), 4.93 – 4.88 (m, 1H), 4.42 – 4.30 (m, 1H), 4.09 – 3.93 (m, 1H), 3.54 – 3.40 (m, 2H), 2.99 (ddd, *J* = 15.8, 11.4, 4.0 Hz, 1H), 2.83 – 2.76 (m, 1H), 1.54 – 1.36 (m, 6H). **^{13}C NMR** (126 MHz, CD_3OD) δ 157.73, 156.81, 156.07, 153.54, 151.57, 137.22, 129.76, 129.71, 127.56, 121.88, 118.67, 117.47, 114.76, 113.72, 111.05, 108.93, 106.46, 102.36, 77.40, 70.19, 65.59, 59.00, 31.65, 30.42, 24.13, 23.91.

11.2 NMR and HR-MS data

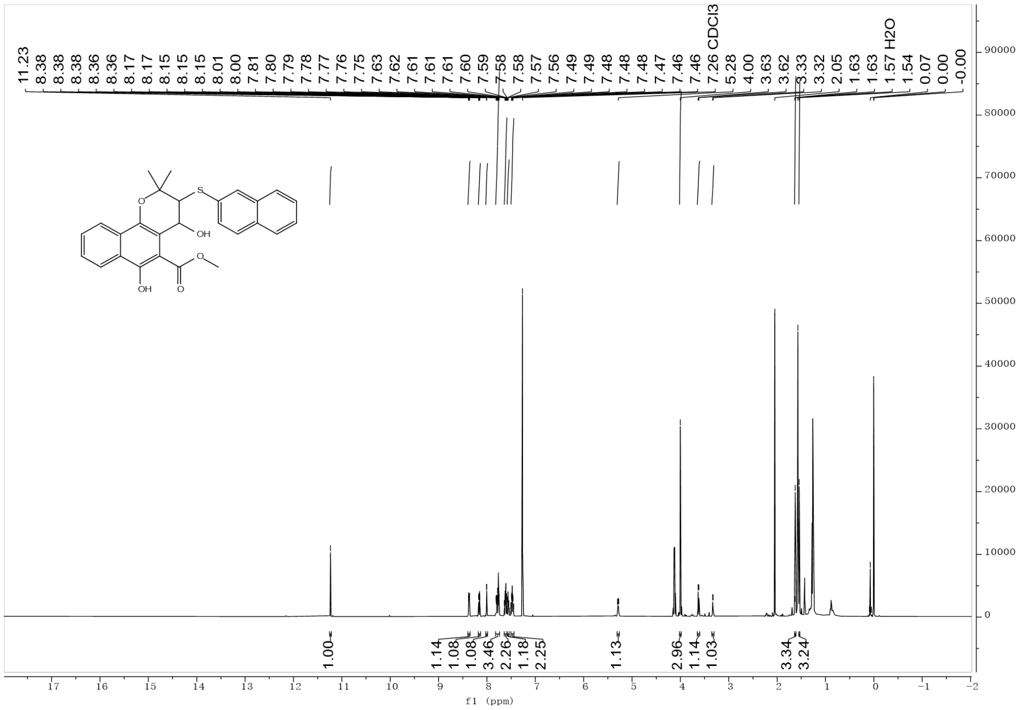

Figure S22. ^{1}H NMR spectrum of compound 7 in $CDCl_3$

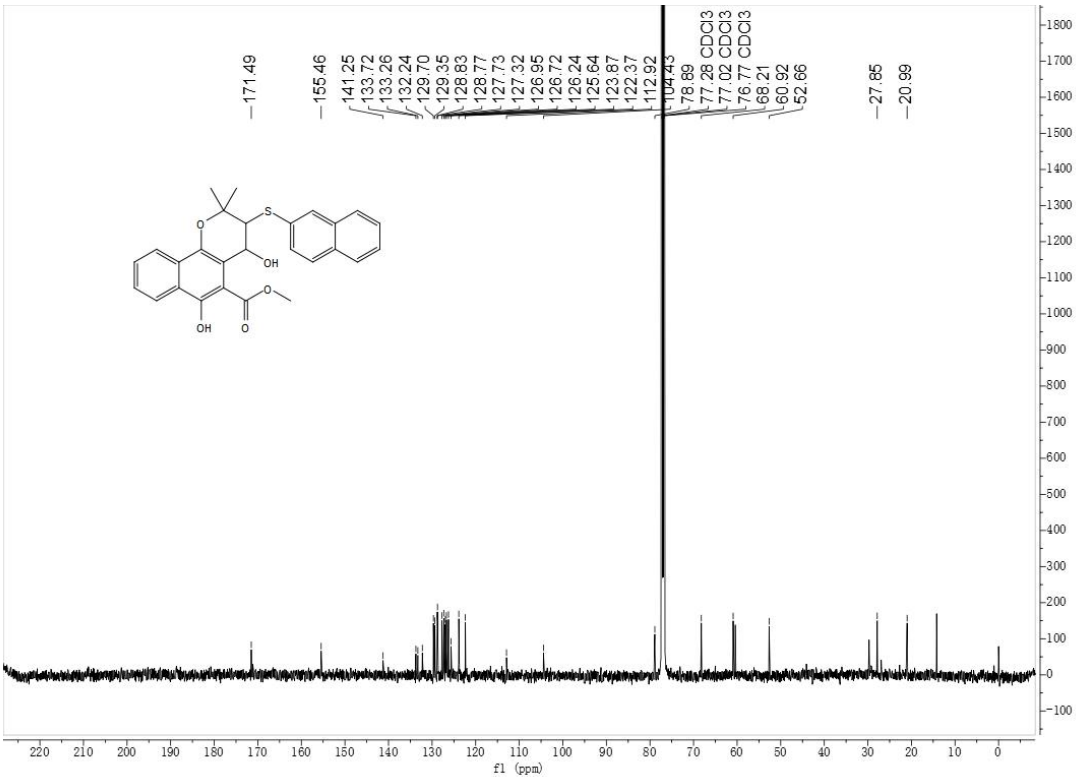

Figure S23. ^{13}C NMR spectrum of compound 7 in $CDCl_3$

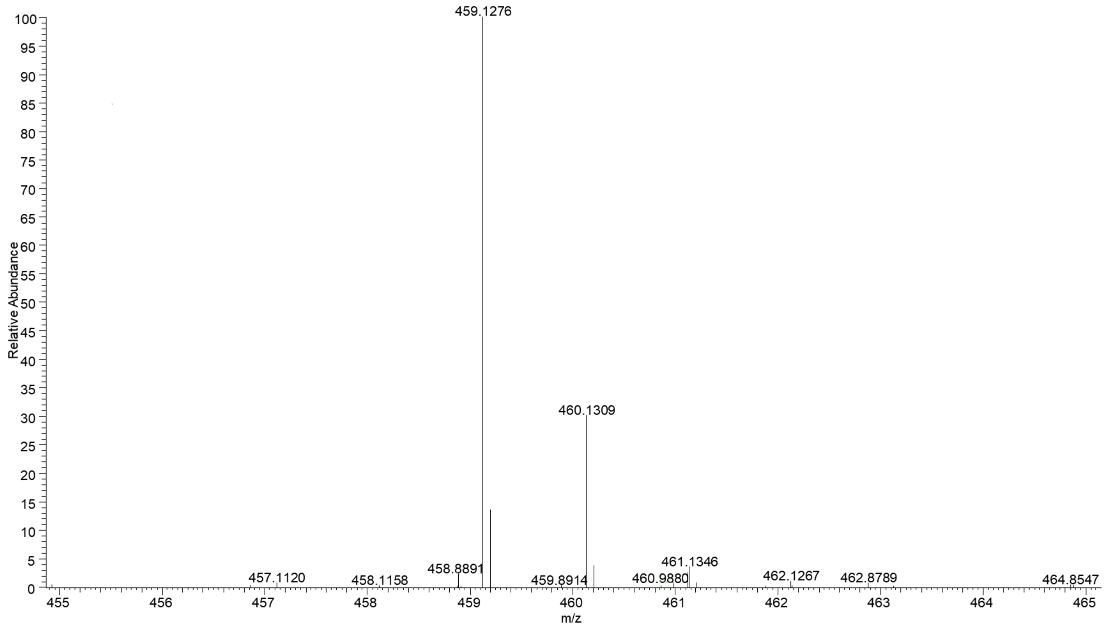

Figure S24. HR-MS spectrum of compound 7

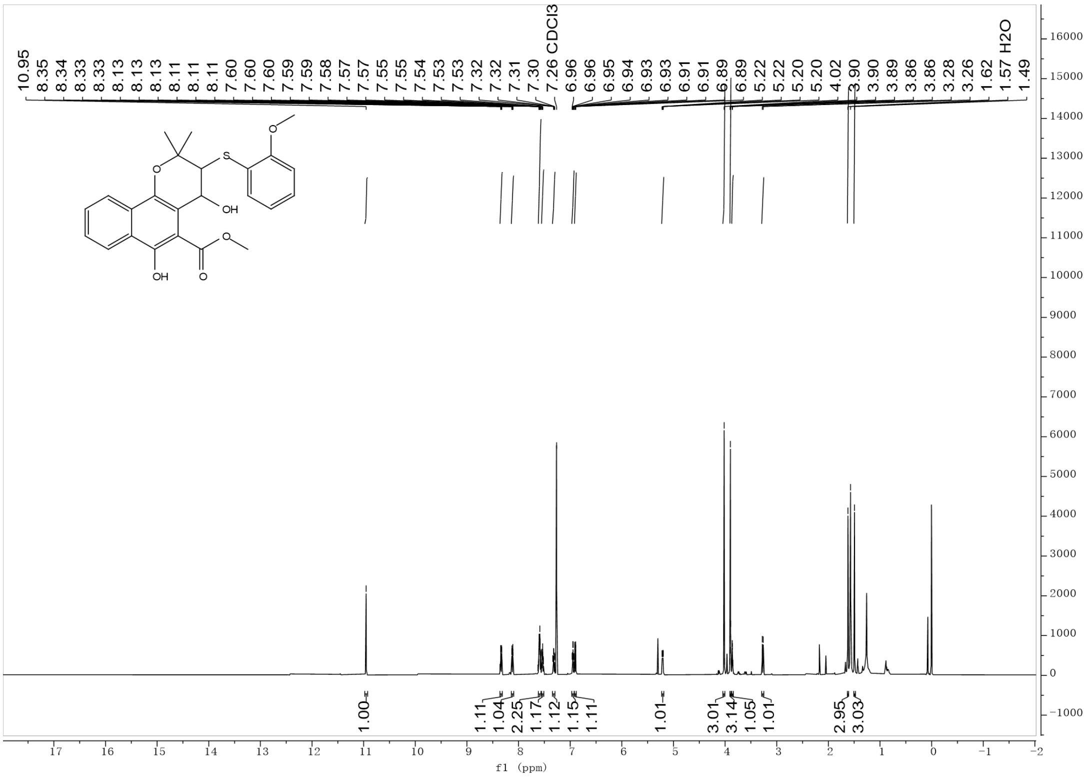

Figure S25. ^{1}H NMR spectrum of compound 8 in $CDCl_3$

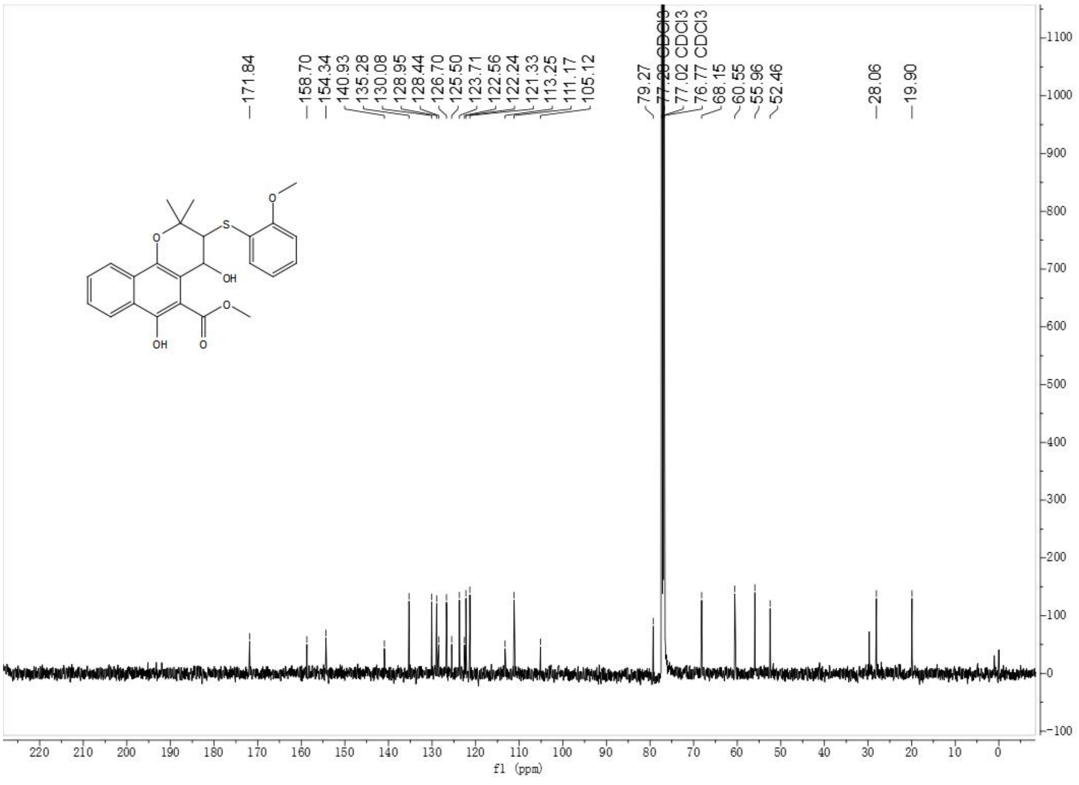

Figure S26. ^{13}C NMR spectrum of compound 8 in $CDCl_3$

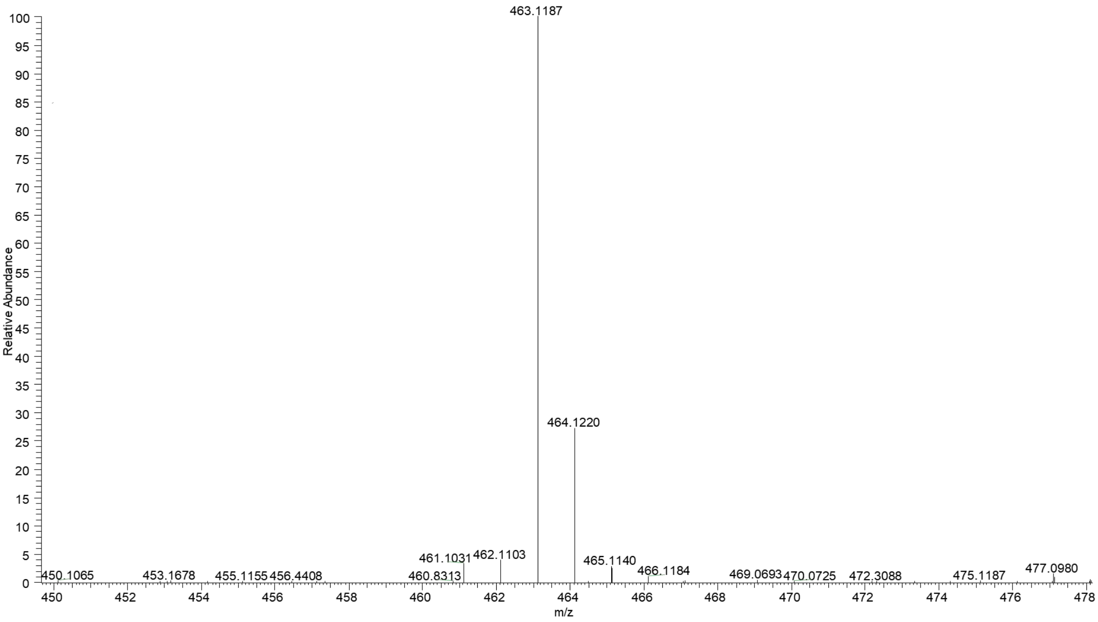

Figure S27. HR-MS spectrum of compound 8

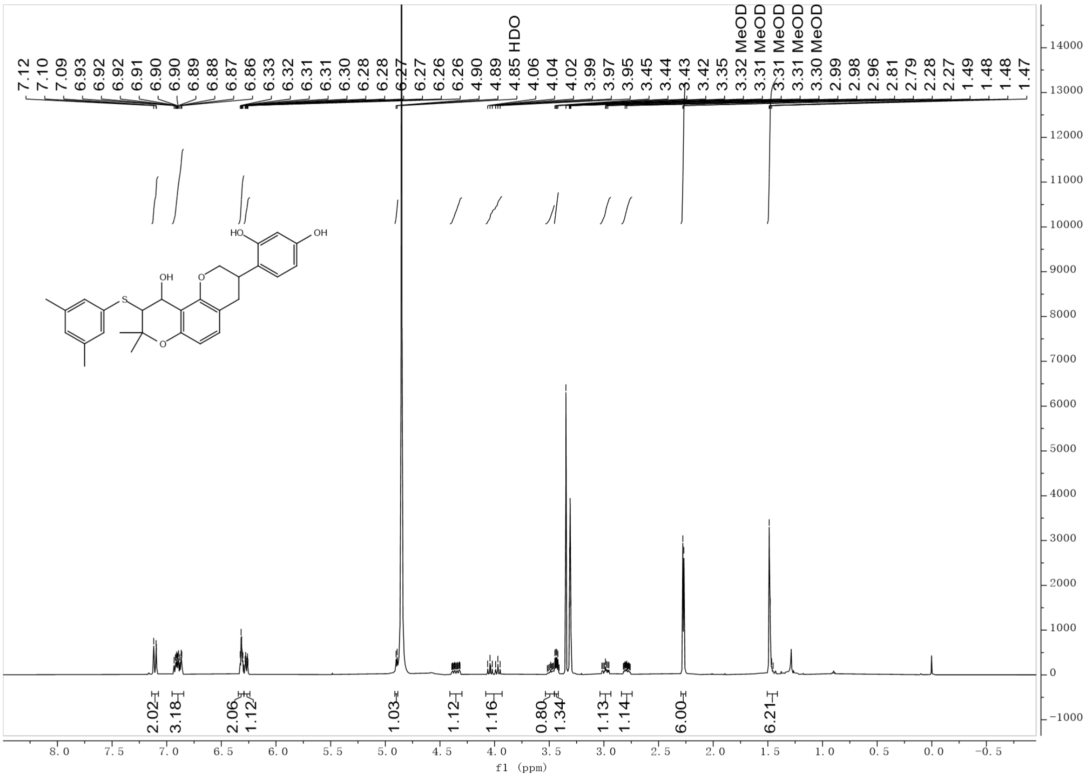

Figure S28. ^{1}H NMR spectrum of compound 13 in CD_3OD

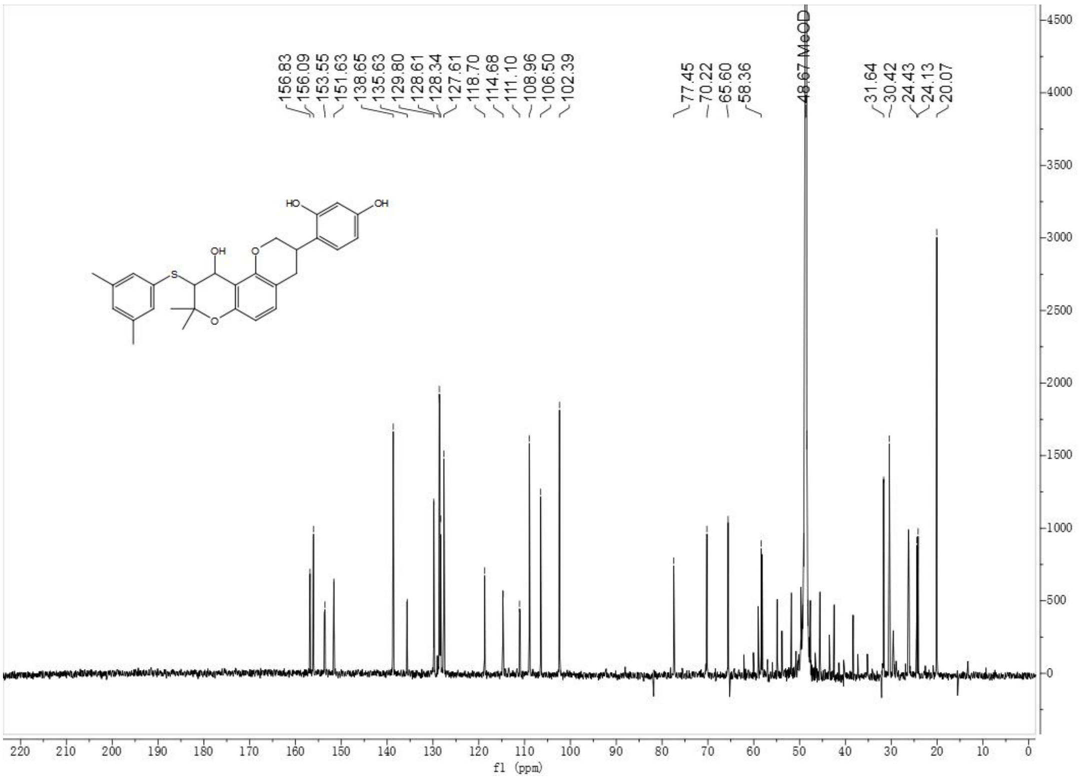

Figure S29. ^{13}C NMR spectrum of compound 13 in CD_3OD

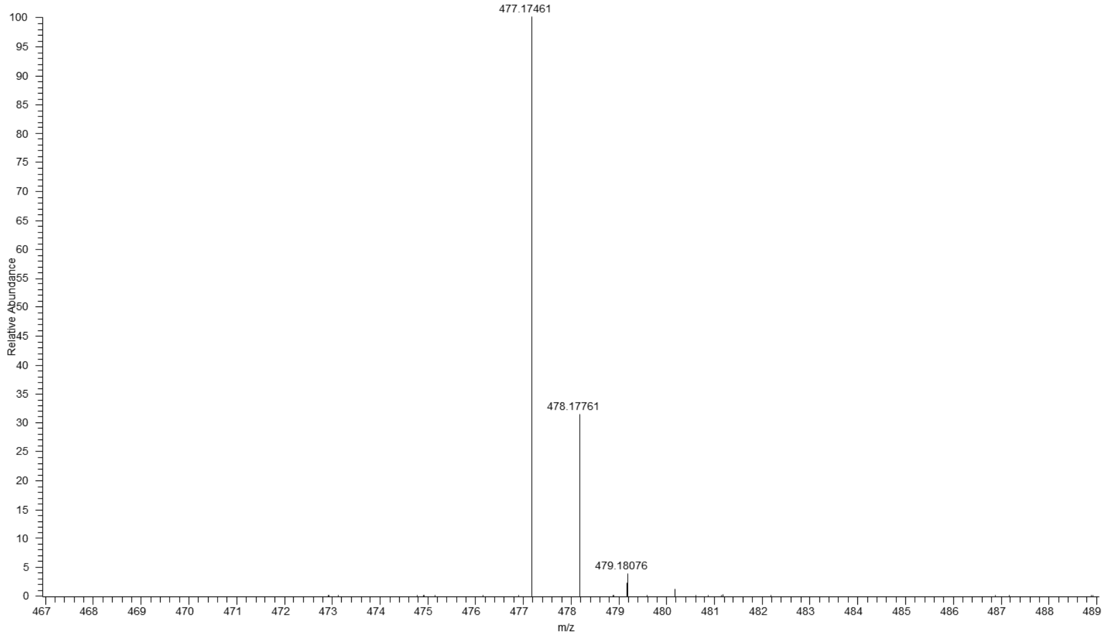

Figure S30. HR-MS spectrum of compound 13

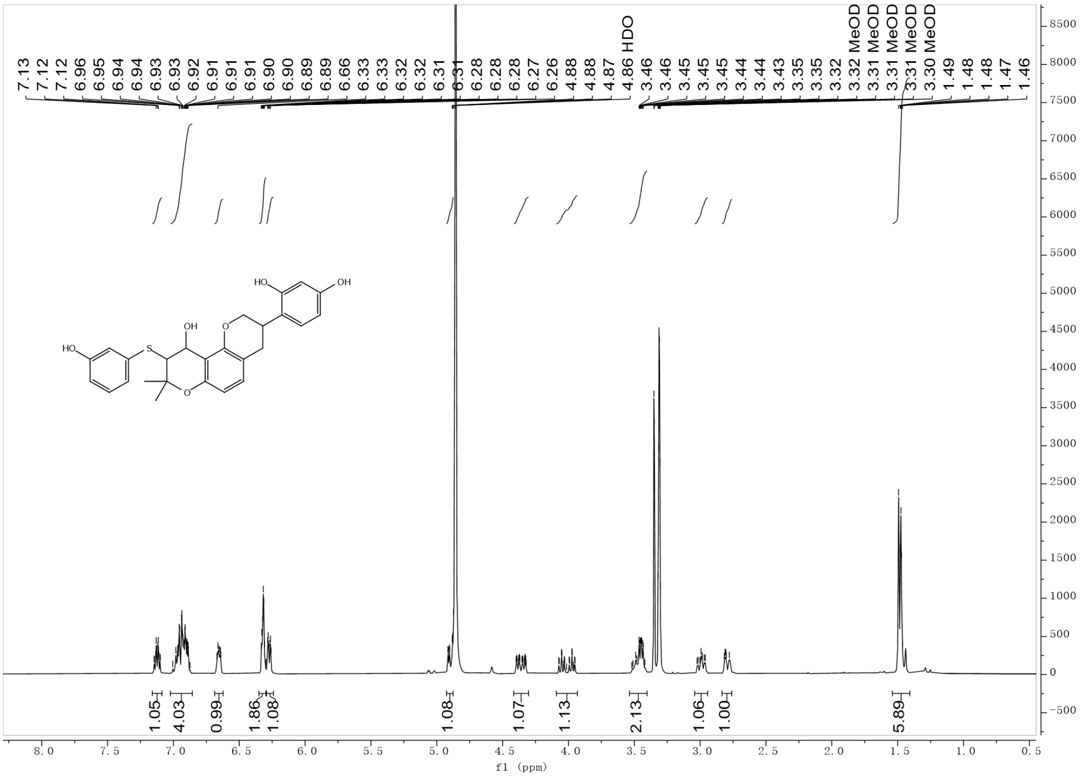

Figure S31. ^{1}H NMR spectrum of compound 14 in CD_3OD

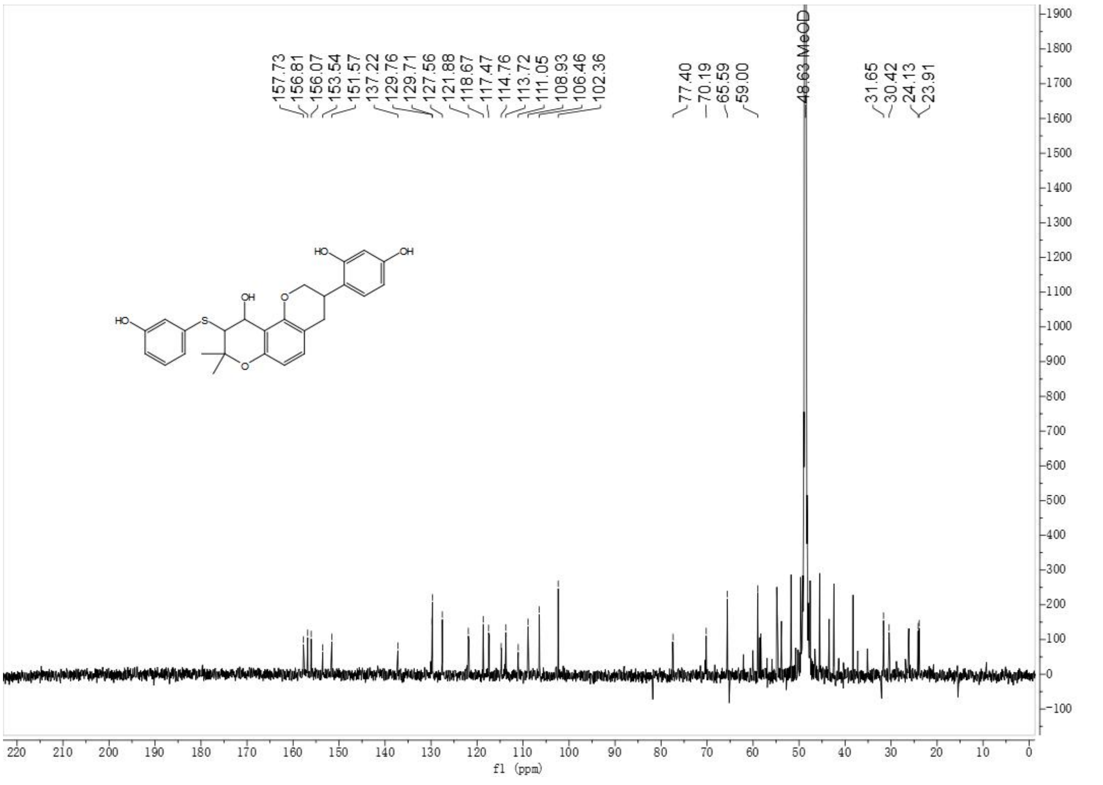

Figure S32. ^{13}C NMR spectrum of compound 14 in CD_3OD

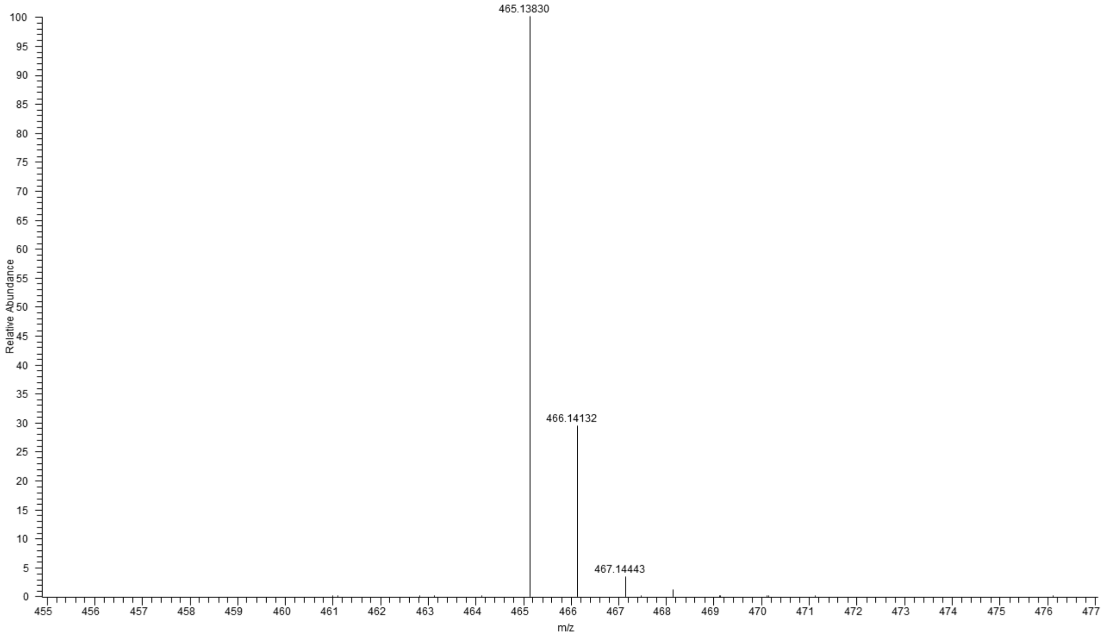

Figure S33. HR-MS spectrum of compound 14
